# A Randomised Controlled Trial of Intravenous Zoledronic Acid in Malignant Pleural Disease: A Proof of Principle Pilot Study

**DOI:** 10.1371/journal.pone.0118569

**Published:** 2015-03-17

**Authors:** Amelia O. Clive, Clare E. Hooper, Anthony J. Edey, Anna J. Morley, Natalie Zahan-Evans, David Hall, Iain Lyburn, Paul White, Jeremy P. Braybrooke, Iara Sequeiros, Stephen M. Lyen, Tim Milton, Brennan C. Kahan, Nick A. Maskell

**Affiliations:** 1 Academic Respiratory Unit, University of Bristol, Bristol, United Kingdom; 2 North Bristol Lung Centre, Southmead Hospital, North Bristol NHS Trust, Bristol, United Kingdom; 3 Cobalt Health, Cheltenham, United Kingdom; 4 Department of Statistics, University of West of England, Bristol, United Kingdom; 5 Bristol Cancer Institute, University Hospitals Bristol NHS Foundation Trust, Bristol, United Kingdom; 6 Department of Oral and Maxillofacial Surgery, University Hospitals Bristol NHS Foundation Trust, Bristol, United Kingdom; 7 Pragmatic Clinical Trials Unit, Queen Mary University of London, London, United Kingdom; National Cancer Centre, SINGAPORE

## Abstract

**Introduction:**

Animal studies have shown Zoledronic Acid (ZA) may diminish pleural fluid accumulation and tumour bulk in malignant pleural disease (MPD). We performed a pilot study to evaluate its effects in humans.

**Methods:**

We undertook a single centre, double-blind, placebo-controlled trial in adults with MPD. Patients were randomised (1:1) to receive 2 doses of intravenous ZA or placebo, 3 weeks apart and were followed-up for 6 weeks. The co-primary outcomes were change in Visual Analogue Scale (VAS) score measured breathlessness during trial follow-up and change in the initial area under the curve (iAUC) on thoracic Dynamic Contrast Enhanced Magnetic Resonance Imaging (DCE-MRI) from randomisation to week 5. Multiple secondary endpoints were also evaluated.

**Results:**

Between January 2010 and May 2013, 30 patients were enrolled, 24 randomised and 4 withdrew after randomisation (1 withdrew consent; 3 had a clinical decline). At baseline, the ZA group were more breathless, had more advanced disease on radiology and worse quality of life than the placebo group. There was no significant difference between the groups with regards change in breathlessness (Adjusted mean difference (AMD) 4.16 (95%CI −4.7 to 13.0)) or change in DCE-MRI iAUC (AMD −15.4 (95%CI −58.1 to 27.3). Two of nine (22%) in the ZA arm had a >10% improvement by modified RECIST (vs 0/11 who received placebo). There was no significant difference in quality of life measured by the QLQ-C30 score (global QOL: AMD -4.1 (-13.0 to 4.9)), side effects or serious adverse event rates.

**Conclusions:**

This is the first human study to evaluate ZA in MPD. The study is limited by small numbers and imbalanced baseline characteristics. Although no convincing treatment effect was identified, potential benefits for specific subgroups of patients cannot be excluded. This study provides important information regarding the feasibility of future trials to evaluate the effects of ZA further.

**Trial Registration:**

UK Clinical Research Network ID 8877 ISRCTN17030426 www.isrctn.com

## Introduction

Malignant pleural disease signifies incurable malignancy and management options are limited. Symptomatic malignant pleural effusion (MPE) may be managed with drainage procedures and/or pleurodesis, but currently available treatment strategies do not target the underlying problem of excessive pleural fluid production. Additionally, patients may be too frail to undergo tumour specific chemotherapy and hence treatment is often supportive.

Zoledronic Acid (ZA) is an intravenous aminobisphosphonate, which is currently licensed for prevention of skeletal related events in adults with advanced cancer involving bone and treatment of tumour-induced hypercalcaemia. It has well documented anti-tumour, anti-inflammatory and anti-angiogenic effects [1, 2]. These properties are appealing in malignant pleural disease as they would target both the underlying tumour and the uncontrolled production of pleural fluid. Exploratory in vivo and in vitro animal studies evaluating ZA in malignant pleural disease have also shown encouraging results [3–7].

Along with its favourable safety profile, allowing ZA to be used in patients otherwise unfit for chemotherapy, this makes it an attractive potential treatment for patients with malignant pleural disease.

The aim of this study was to generate pilot clinical data regarding the efficacy of ZA in malignant pleural disease in order to inform a future, suitably powered randomised controlled trial should the results show promise.

## Materials and Methods

The protocol for this trial and supporting CONSORT checklist are available as supporting information; see S1 CONSORT Checklist and S1 Protocol.

### Trial Design

The Bristol Randomised Trial of Zoledronic Acid in Malignant Pleural Disease (Pilot Study) was a double-blind, placebo controlled, randomised trial. Ethical and regulatory approval for the study was obtained from the South West Research Ethics Committee (REC 09/H0206/12) and the Medicines and Healthcare products Regulatory Agency (MHRA EudraCT 2009-009134-32) before recruitment commenced. After written informed consent, patients were randomised to receive 2 doses of intravenous ZA or placebo 3 weeks apart.

### Participants Enrolled

Adults with malignant pleural thickening (with or without pleural effusion) were enrolled into the study. The diagnosis of malignant pleural disease required positive pleural fluid cytology and/or pleural biopsy, or a clinically confident diagnosis of MPE in the context of proven cancer elsewhere.

The exclusion criteria were: Pleurodesis in the preceding 30 days; iv bisphosphonates administered within the past 3 months; ongoing dental disease; significant renal impairment (calculated creatinine clearance (CrCl) <40ml/min); hypocalcaemia; inability to give informed consent; pregnancy or lactation; known allergy to bisphosphonates or its excipients; life expectancy <4 months; current or planned chemotherapy; hormone manipulation therapy initiated in the month prior to trial entry; age <18 years; haematological malignancy; severe visual impairment.

Minor alterations made to the eligibility criteria during the recruitment period are detailed in the online S1 Protocol.

Patients were identified from the pleural and oncology clinics and the lung cancer multi-disciplinary team (MDT) meeting at North Bristol NHS Trust. Eligible patients were provided with a patient information sheet (see online S1 File: Patient information sheet) and written consent was obtained.

### Randomisation

Patients were randomised 1:1 to either ZA or placebo using minimisation. A random element was used, so that patients were assigned to the treatment arm which minimised the imbalance with a 95% probability. The minimisation factors were: the presence or absence of trapped lung; tumour type (mesothelioma/ other); and the presence or absence of an indwelling pleural catheter (IPC). Randomisation was performed using a centralised online randomisation service (Sealed Envelope, London, UK).

### Blinding

Patients, clinicians and trial investigators were blind to treatment allocation. The ZA and placebo preparations were indistinguishable. The radiology and biological samples were analysed and the statistical analysis plan finalised prior to un-blinding.

### Trial Interventions

The full details of the study protocol are available in the online S1 Protocol. After written consent (see S2 File: Consent form), patients had a 2 week run-in period to confirm study eligibility and undergo baseline assessments. All patients underwent a dental check and any necessary dental treatment during the run-in period to minimise the risk of osteonecrosis of the jaw (as per the guidance in the summary of product characteristics for Zoledronic Acid).

The dose of ZA was calculated according to their estimated CrCl (CrCl ≥60ml/min: 4mg ZA; CrCl ≥50-<60ml/min: 3.5mg ZA; CrCl ≥40-<50ml/min: 3.3mg ZA) and provided by the pharmacy in 100ml 0.9% sodium chloride. The placebo was 100ml 0.9% sodium chloride. A second dose of Investigational Medicinal Product (IMP) was administered 3 weeks later.

All participants received other treatments during trial follow up in accordance with standard care guidelines.

### Trial Assessments

Patients were followed for 6 weeks after randomisation. Patients completed a daily Visual Analogue Scale (VAS) score assessing breathlessness for a week prior to randomisation until the end of trial follow-up. The European Organisation for Research and Treatment of Cancer Quality of Life Questionnaire (EORTC-QLQ-C30) [8], the Edmonton Symptom Assessment System (ESAS) QOL questionnaire, Medical Research Council (MRC) dyspnoea score and thoracic ultrasound (USS) were completed at randomisation, week 3 and week 6. A CT and MRI thorax were performed in the week prior to randomisation and during week 5 (see S1 Protocol, S3 File and S4 File: SOP for DCE MRI analysis for scan protocols). Patients with an indwelling pleural catheter (IPC) underwent twice weekly drainages and fluid was stored at −80°C for future analysis (see S3 File). Blood tests were performed on a weekly basis for monitoring purposes and samples were stored at −80°C for future analysis (see S3 File).

### Trial Outcomes

The primary endpoints were: (1) Change from baseline to week 5 in the ‘initial area under the curve measurement for the first 90 seconds’ (iAUC) of the DCE-MRI scan and (2) The summary score for the dyspnoea VAS score from randomisation to week 6 post-randomisation.

The main objective of this pilot trial was to assess potential efficacy of ZA in this patient group to inform a future, suitably powered RCT. Therefore numerous secondary clinical and feasibility outcomes were evaluated over the 6 week trial follow up (see S5 file: Statistical analysis plan for full details). This included changes from baseline to week 5 in Dynamic Contrast Enhanced MRI (DCE-MRI) and Diffusion weighted MRI (DWI-MRI) parameters; modified response evaluation in solid tumours (RECIST) scores; CT measured pleural thickening and effusion volumes. Also, changes in USS effusion depths; quality of life and symptom control (using the EORTC QLQ-C30, ESAS score and Medical Research Council (MRC) dyspnoea scale) and blood parameters (serum mesothelin, plasma VEGF-A, plasma IL-6, MCP-1, serum neutrophil to lymphocyte ratio (NLR)) recorded at baseline, week 3 and week 6 were assessed. For patients with an IPC, weekly pleural fluid VEGF-A and MCP-1 levels and pleural fluid production were also evaluated. Pharmacokinetic analysis of ZA levels in the pleural fluid and pharmacokinetic analysis of the DCE-MRI scans was planned but was unable to be performed due to resource issues.

The CT and MRI scans were reported independently by 2 respiratory radiologists and discrepancies resolved by consensus. Further details on the reporting methods can be found in S1 Protocol, S3 File and S4 File: SOP for DCE MRI analysis.

Safety outcomes were also evaluated up to 6 weeks post-randomisation. This included the number of patients: (1) experiencing at least one known side effect of Zoledronic Acid; (2) experiencing at least one serious adverse event; and (3) requiring an increase in calcium, magnesium or phosphate replacement [9]. The side effects were categorized based on the participants’ case report forms and blood results prior to un-blinding of the patients’ treatment allocations.

### Statistical Methods

Data was analysed on intention-to-treat principles. All randomised patients in whom an outcome was available were included in the analysis. Data collected up to the point of patient withdrawal were included. All analyses were pre-determined prior to data analysis and un-blinding. All analyses adjusted for outcome value measured at baseline (if recorded) and the minimisation factors (the presence or absence of trapped lung; tumour type (mesothelioma/ other); the presence or absence of an IPC) [10–12]. Mean imputation was used to account for baseline variables with missing data [13]. All outcomes were analysed using a linear regression model except for the main adverse event outcomes, which were analysed using logistic regression. The linear regression models provided an adjusted mean difference (AMD) to quantify the treatment effect. The linear regression models reported treatment effect as Odds Ratios.

Outcomes which were collected at multiple time points during trial follow up (VAS scores, QOL measures, blood and pleural fluid biomarkers, pleural fluid production and USS depths) were analysed using a ‘summary score’, which was calculated by dividing the area under the curve (AUC) by the number of days that the patient remained in the study (to account for different durations of follow-up). The AUC was calculated using the trapezium rule [14].

Stata software version 13 (Texas, USA) was used for analyses. Full details of the statistical analysis plan are in the online S5 File: Statistical analysis plan.

The trial was registered with the MHRA (EudraCT 2009-009134-32) and the UK Clinical Research Network (UKCRN ID 8877) prior to recruitment commencing. It was subsequently also registered with an International Standard Randomised Controlled Trial Number ISRCTN17030426.

### Sample Size Calculation

The study was designed as a proof of principle, pilot study and was the first time ZA had been used in humans for this indication. Therefore, a power calculation was not feasible. However, a target recruitment of 40 patients was chosen and subsequently reduced to 30 due to difficulties with recruitment.

### Trial Funding

This trial was supported by unrestricted research grants from North Bristol NHS Trust (small grants scheme), Novartis and UK Medical. The study was sponsored by North Bristol NHS Trust. The IPCs and drainage bottles were provided by UK Medical. The funders had no role in study design, data collection and analysis, decision to publish or preparation of the manuscript.

## Results

### Feasibility Outcomes

**Fig 1:**
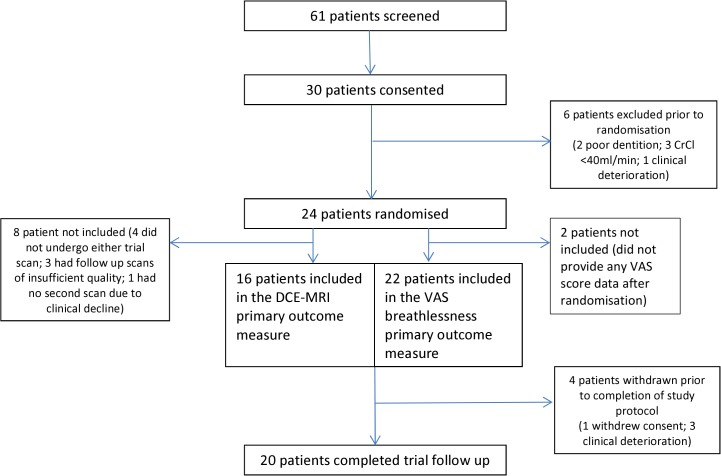
Patient flow diagram.

The patient flow diagram is shown in Fig. 1. The trial was granted ethics approval on 18/05/2009. 30/61 screened patients (49%) were recruited from North Bristol NHS Trust between 5^th^ January 2010 and 8^th^ May 2013. The final patient completed trial follow up on 2^nd^ July 2013. 24/30 consented patients (80%) were subsequently randomised and 20 patients completed the trial follow up. 11/30 consented patients were found to have poor dentition at their baseline dental assessment, 2 of whom were withdrawn from the study and 9 required dental intervention prior to ongoing trial involvement. Reasons for the exclusions and withdrawals are given in Fig. 1.

Two of the 24 randomised patients did not receive any doses of study treatment (1 patient withdrew consent, 1 patient withdrawn due to clinical decline) and 2 patients received only one dose (1 patient declined second dose; 1 patient withdrawn prior to 2^nd^ dose due to clinical decline). The remaining 20 patients (83%) received both doses (see online S3 File).

2/11 patients who received ZA required a dose reduction (1 due to low CrCl and 1 due to ‘flu-like symptoms after the first dose).

### Patients

Baseline characteristics are presented in Table 1. There were a number of imbalances between the study arms. There were more males in the placebo group compared to the ZA group (85% vs 45%). The placebo group were less breathless than the ZA group at randomisation, as measured by the MRC dyspnoea score (2.3 (SD 1.1) vs 3.1 (SD 1.1)), dyspnoea VAS score (22.9mm (SD 12.3) vs 33.5mm (SD 15.2)) and QLQ-C30 dyspnoea score (27.8 (SD 13.0) vs 56.7 (SD 22.5)).

**Table 1:** Baseline characteristics of the randomised patients.

	Placebo (n = 13)	Zoledronic Acid (n = 11)
Male, n (%)	11 (85)	5 (45)
Age, mean (SD)	72.6 (9.9)	71.2 (8.3)
Side		
Left, n (%)	4 (31)	4 (36)
Right, n (%)	9 (69)	6 (55)
Bilateral, n (%)	0	1 (9)
Cell type		
Mesothelioma, n (%)	7 (54)	7 (64)
Lung Cancer, n (%)	3 (23)	3 (27)
Other, n (%)	3 (23)	1 (9)
Presence of malignant pleural effusion, n (%)	11 (85)	8 (73)
Mode of diagnosis		
Pleural biopsy, n (%)	8 (62)	7 (64)
Pleural fluid cytology, n (%)	4 (31)	3 (27)
Other, n (%)	1 (8)	1 (9)
Time from diagnosis to trial entry, days, median (IQR)	180 (105–293)	205 (48–241)
Previous chemotherapy	6 (46)	7 (64)
On steroids at trial entry, n (%)	2 (15)	1 (9)
Previous talc pleurodesis, n (%)	4 (31)	2 (18)
Previous thoracic surgery, n (%)	1 (8)	2 (18)
IPC in situ at trial entry, n (%)	4 (31)	2 (18)
Trapped lung, n (%)	3 (23)	2 (18)
Dental treatment required in study run-in period, n (%)	3 (25)	6 (55)
Imaging		
DCE-MRI ‘area under the curve in the first 90s’ (iAUC), mean (SD)	113.9 (49.9)	128.1 (57.6)
Modified RECIST score, mean (SD)	55.6 (30.3)	63.9 (34.2)
Summed ultrasound effusion depth, mean (SD)	8.6 (11.5)	5.0 (5.8)
Blood tests		
Serum WCC, mean (SD)	11.3 (10.4)	8.3 (3.2)
Haemoglobin, mean (SD)	12.7 (1.7)	12.7 (2.1)
Platelets, mean (SD)	280 (102)	345 (126)
Creatinine clearance, mean (SD)	72.6 (17.1)	81.6 (19.4)
Corrected calcium, mean (SD)	2.4 (0.2)	2.4 (0.1)
Phosphate, mean (SD)	1.2 (0.2)	1.1 (0.2)
Magnesium, mean (SD)	0.79 (0.08)	0.80 (0.06)
NLR, mean (SD)	6.0 (6.6)	4.0 (3.6)
Quality of life		
EORTC QLQC30- global quality of life, mean (SD)	67.4 (20.6)	55.8 (11.8)
EORTC QLQC30- physical functioning score, mean (SD)	72.8 (16.4)	62.7 (19.7)
ESAS score, mean (SD)	15.9 (7.7)	24.1 (9.9)
Dyspnoea Scores		
Dyspnoea VAS score (mm)	22.9 (12.3)	33.5 (15.2)
Bother about breathlessness VAS score	23.0 (13.1)	31.6 (14.6)
MRC dyspnoea score	2.3 (1.1)	3.1 (1.1)
EORTC QLQC30- dyspnoea score, mean (SD)	27.8 (13.0)	56.7 (22.5)

SD = Standard Deviation; IPC = Indwelling pleural catheter; DCE-MRI = Dynamic Contrast Enhanced Magnetic Resonance Imaging; RECIST = Response evaluation criteria in solid tumours; WCC = white cell count; NLR = Neutrophil to Lymphocyte ratio; EORTC QLQ-C30 = European Organisation for Research and Treatment of Cancer Quality of Life Questionnaire; ESAS = Edmonton Symptom Assessment Scale; VAS = Visual Analogue Scale; MRC = Medical Research Council.

The placebo group also had a better baseline QOL compared to the ZA group, with a mean QLQ-C30 overall QOL score of 67.4 (SD 20.6) and 55.8 (SD 11.8), a mean QLQ-C30 physical functioning score of 72.8 (SD 16.4) and 62.7 (SD 19.7) respectively and a mean ESAS score of 15.9 (SD 7.7) and 24.1 (SD 9.9) respectively.

Additionally, at randomisation the placebo group had less advanced disease on radiology than the ZA group, with a mean baseline modified RECIST score of 55.6 (SD 30.3) and 63.9 (SD 34.2) respectively. The placebo group also had a lower baseline DCE-MRI iAUC at baseline than the ZA group (113.9 (SD 49.9) vs 128.1 (57.6) respectively).

There were a number of other small differences between the treatment groups at baseline, although the relevance of these in terms of any potential treatment effect is not clear. A smaller proportion of patients in the placebo group had received previous chemotherapy (6/13 vs 7/11 in the ZA group). More patients randomised to placebo had undergone a talc pleurodesis in the past (4/13 vs 2/11 in the ZA group) and more were taking steroids at trial entry (2/13 vs 1/11 in the ZA group).

No patients received any other systemic treatments for cancer during their trial involvement.

### Primary Outcomes

#### VAS Breathlessness Score

22/24 randomised patients were included in the analysis of the breathlessness VAS Score (1 withdrew consent; 1 provided no VAS score data post randomisation). As described above, patients in the ZA group had higher baseline VAS breathlessness scores than those in the placebo arm.

**Fig 2:**
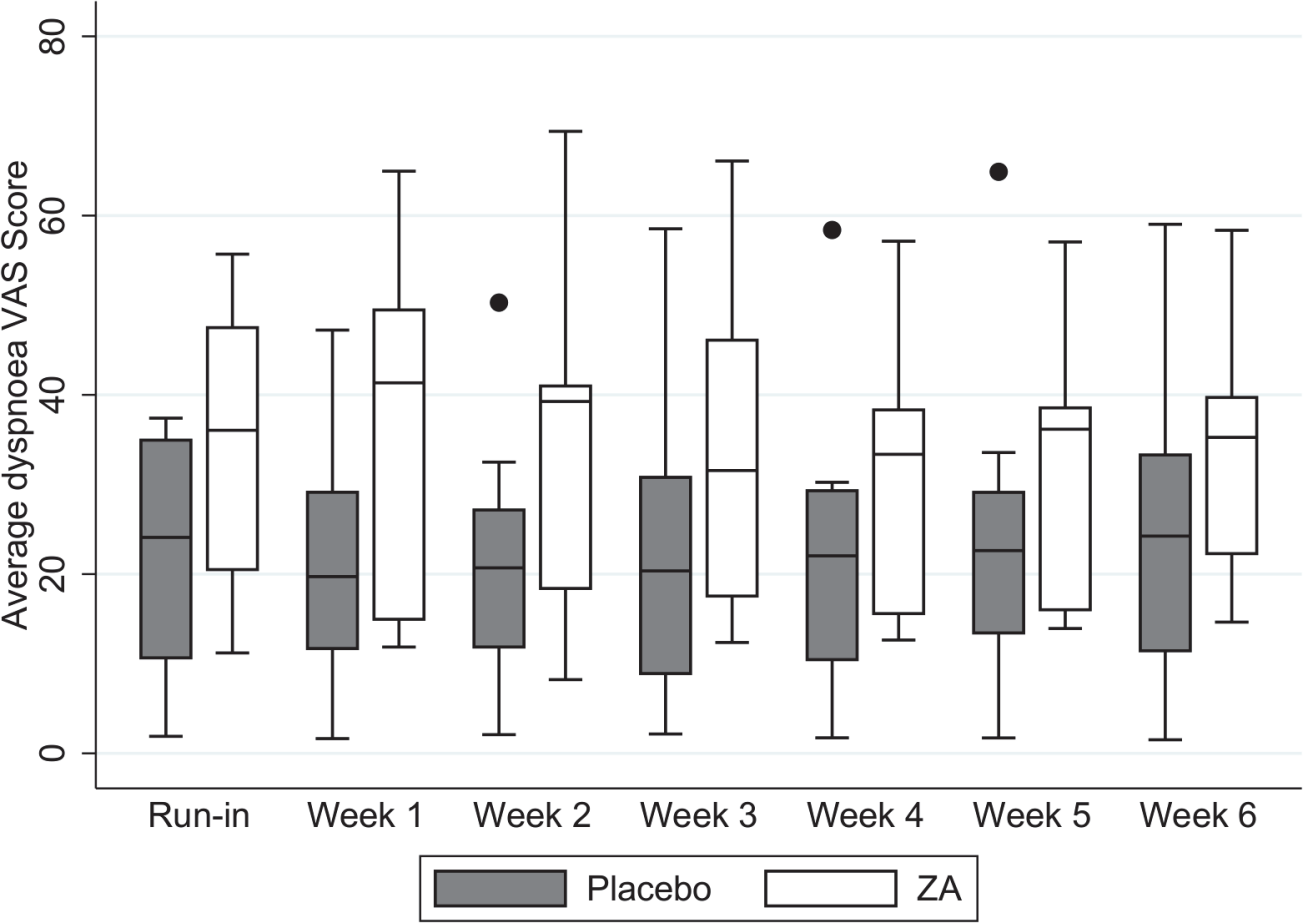
Box and whisper plot evaluating the average weekly VAS breathlessness scores for the 2 treatment arms. The horizontal line with the box represents the median. The box edges represent the lower (25^th^) and upper (75^th^) quartiles. The whiskers represent the lower and upper adjacent values. Outside values are plotted separately.

There was no significant difference in the summary scores for the breathlessness VAS score between treatment arms. The VAS summary score was 21.6 (SD 13.4; n = 12) in the placebo arm compared with 34.0 (SD 15.0; n = 10) in the ZA group (adjusted mean difference (AMD) 4.2 (95%CI −4.7 to 13.0)). The change in mean VAS breathlessness score over time for the two study arms is shown in Fig. 2.

There were insufficient patients with an IPC in-situ, trapped lung or who initiated chemotherapy or endocrine therapy during trial follow up to allow meaningful subgroup analyses. Subgroup analysis of the mesothelioma patients did not show any important differences.

#### DCE-MRI

**Fig 3:**
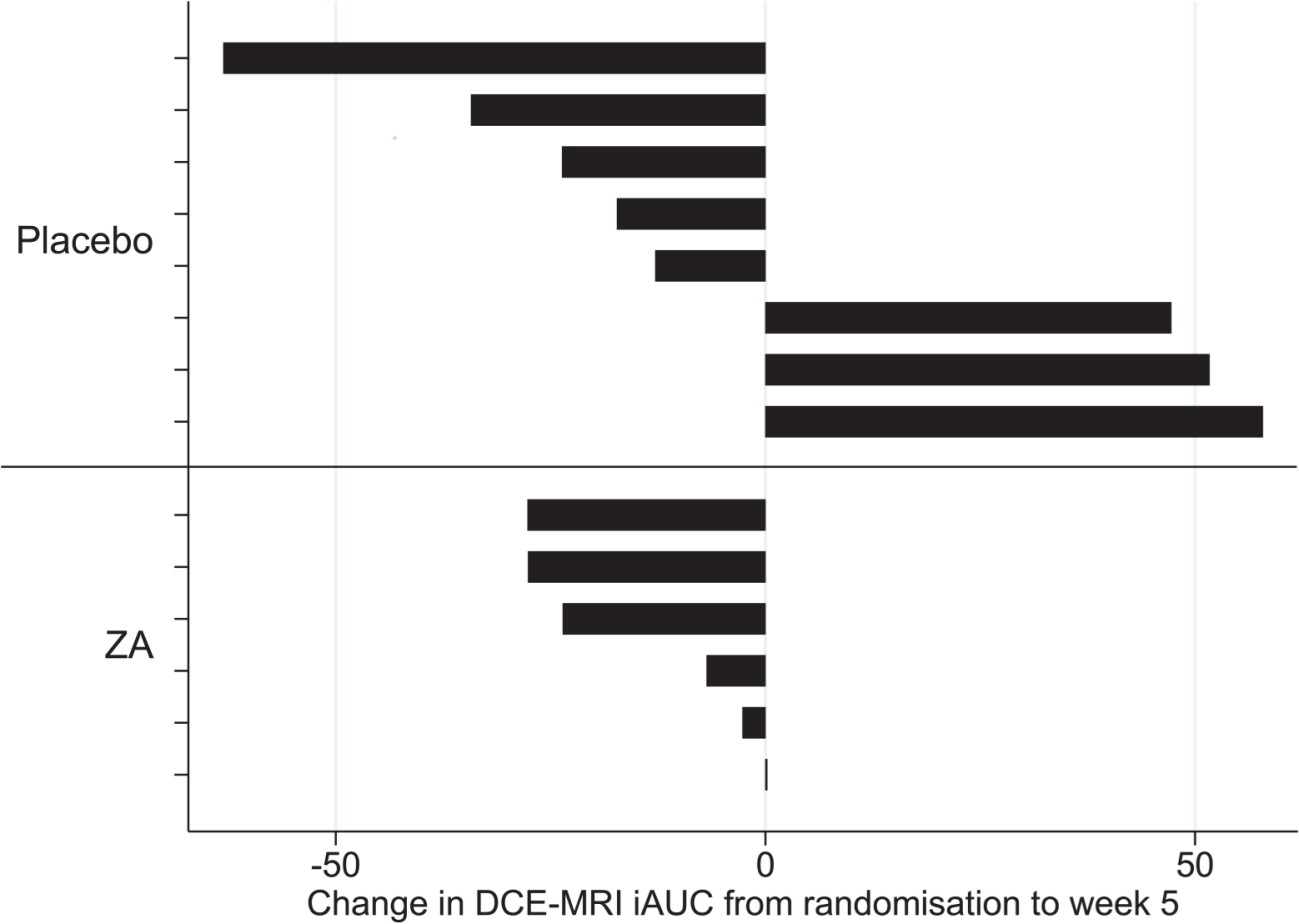
Change in DCE iAUC from baseline to week 5 (complete case data).

16/24 randomised patients were included in the DCE-MRI analysis (4 patients did not undergo either trial MRI scan; 3 had follow-up MRIs of insufficient quality due to movement artefact; 1 had no second scan due to a clinical decline). There was no significant difference in the change in the iAUC from baseline to week 5 in the placebo group vs the ZA group (7.3 (SD 46.8; n = 9) and -20.9 (SD 20.4; n = 7) respectively; AMD -15.4 (95% CI −58.1 to 27.3)). This trend towards an improvement in perfusion in the ZA arm is likely to be due to test-to-test variability rather than a true response, given the relative improvement in values for some patients in both arms (see Fig. 3).

There were insufficient numbers to meaningfully perform the predefined subgroup analyses.

### Secondary Outcomes

#### Modified RECIST

21 patients had a baseline modified RECIST score. 6/11 in the placebo arm had measurable disease according to modified RECIST on their baseline scan, compared to 7/10 in the ZA arm.

**Fig 4:**
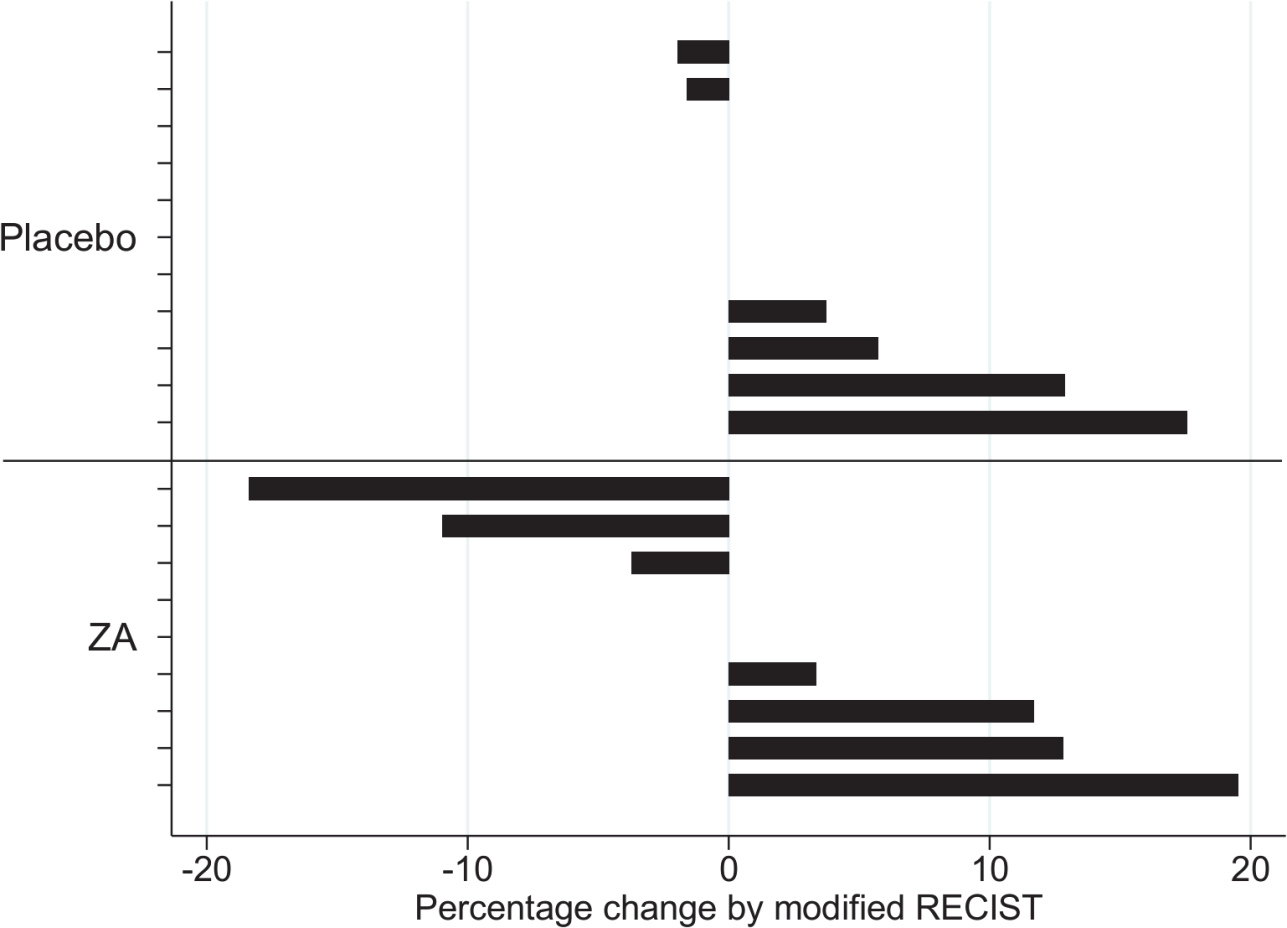
% Change in modified RECIST scores from baseline to week 5.

All 20 evaluable patients in the study had stable disease according to the modified RECIST criteria. However, 2 patients in the ZA group had a >10% reduction in their modified RECIST score from baseline to week 5, compared with none in the placebo group (one patient with adenocarcinoma of unknown primary had a 9mm (18%) improvement; one with mesothelioma had an 8mm (11%) improvement) (see Fig. 4).

The mean change in the modified RECIST scores between the groups was not statistically significant (3.3 (SD 6.1; n = 11) in placebo arm vs 1.7 (SD 7.4; n = 9) in the ZA arm; AMD -1.8 (95%CI −7.9 to 4.3)).

#### Breathlessness

All of the other tools used to evaluate patient dyspnoea (MRC dyspnoea score, QLQ-C30 dyspnoea domain and ‘bother’ VAS score) showed similar trends in their results to the primary outcome VAS Score. The ZA group were consistently more breathlessness at baseline and the treatment did not significantly alter breathlessness (see online S3 File: full results).

#### Quality of life

All three of the quality of life tools evaluated (ESAS, QLQ-C30 global QOL domain and QLQ-C30 physical functioning domain) showed consistent results. The ZA group had worse QOL at baseline and there was no significant differences between the groups in terms of the change in QOL during trial follow up (see online S3 File).

#### Dynamic Contrast Enhanced MRI

In addition to the primary endpoint, three other measurements DCE-MRI parameters were evaluated (Change in the time-to-peak enhancement, change in maximal percentage enhancement, change in the initial wash-in slope). None of the DCE-MRI parameters revealed differences between the treatment arms (see online S3 File). Neither patient who displayed an improvement in their modified RECIST scores had interpretable MRI data (MRI contraindicated in one and uninterpretable week 5 images for the other).

#### Diffusion weighted MRI

The numbers of patients with evaluable DWI-MRI imaging was small (8/24). There was no significant difference between the 2 treatment groups (see online S3 File).

#### Biomarkers

**Fig 5:**
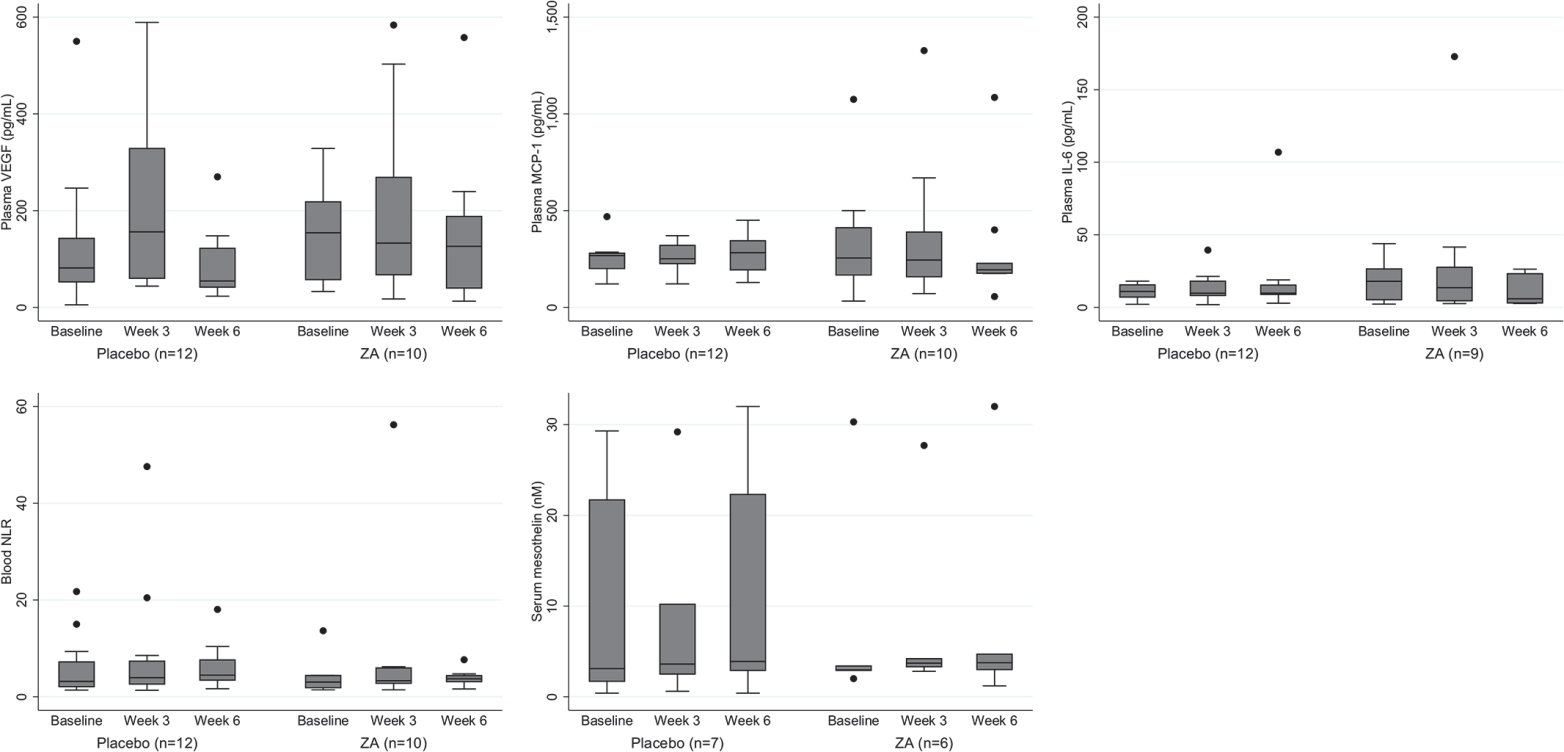
Box and whisper plot showing the change in blood biomarkers over time for the 2 treatment arms. The horizontal line with the box represents the median. The box edges represent the lower (25^th^) and upper (75^th^) quartiles. The whiskers represent the lower and upper adjacent values. Outside values are plotted separately.

Evaluating the changes in serum biomarkers over time (mesothelin, serum VEGF, NLR, IL6 and MCP-1) there was no difference seen between the treatment arms (see Fig. 5 and online S3 File).

#### Pleural fluid volumes

Only 6/24 randomised patients had IPCs in situ (4 in the placebo arm and 2 in the ZA arm), therefore the numbers were too small for formal analysis. No difference was identified in IPC fluid output in these patients (see online S3 File).

Volumetric analysis of the effusion volume on CT (excluding patients with an IPC in situ or those who had undergone a pleural aspiration during trial follow up), showed no significant difference in the change in effusion volume from randomisation to week 5 between the 2 study arms (AMD 10 (95%CI −14 to 34)).

Excluding those patients who underwent a pleural aspiration during trial follow up and those with an IPC in situ, there was no difference between the treatment groups when evaluating the ultrasound summary scores for effusion volume (AMD 0.66 (95%CI −0.53 to 1.85)).

#### Safety

The frequency of adverse events are summarised in Table 2. 21/24 randomised patients experienced at least one side effect and the rate was comparable for the 2 treatment arms (11/13 in placebo arm vs 10/11 in ZA arm; Odds Ratio (OR) 1.8 (95% CI 0.1–23.3)). Evaluating the individual side effects separately, the rates were also comparable between the two arms of the study, although slightly more patients experienced fever or ‘flu like syndrome in the ZA group (4/11 vs 2/13 in the placebo group).

**Table 2:** Side effects and adverse events.

Side Effect	Placebo, number affected (%) (n = 13)	Zoledronic Acid, number affected (%) (n = 11)
Any side effect	11 (85)	10 (91)
Haematological disorder	0 (0)	0 (0)
Rash	0 (0)	1 (9)
Headache	1 (8)	1 (9)
Fever or ‘Flu like syndrome	2 (15)	4 (36)
Myalgia/arthralgia	4 (31)	3 (27)
Renal disturbance	0 (0)	0 (0)
Eye disorder	0 (0)	1 (9)
Gastrointestinal symptoms	6 (46)	5 (45)
Dizziness	2 (15)	3 (27)
Lethargy	4 (31)	4 (36)
Electrolyte disturbance requiring supplementation	3 (23)	4 (36)
Osteonecrosis of the jaw	0 (0)	0 (0)
Serious Adverse Event	5 (38)	3 (27)

8/24 patients experienced a serious adverse event (5/13 in the placebo arm and 3/11 in the ZA arm; OR 0.67 (95% CI 0.11 to 4.08)). Only one was potentially attributable to the study drug (one patient in the ZA arm admitted with flu like symptoms and myalgia 2 days after the first dose of IMP, which resulted in the patient declining the second dose). See online S3 File for further details.

Comparable numbers of patients required an increase in their calcium, magnesium or phosphate replacement during trial follow up (3/13 (23%) in placebo group vs 4/11 (36%) in the ZA group; OR 2.5 (95%CI 0.3 to 19.3)).

## Discussion

This is the first randomised controlled trial to evaluate the effects of Zoledronic Acid in patients with malignant pleural disease. We did not identify any differences in our primary outcome measures, breathlessness and DCE-MRI. However, two patients who received ZA demonstrated a >10% reduction in tumour bulk as assessed by modified RECIST compared to none in the placebo arm. In addition the number and type of side effects were comparable between the two study arms.

There is a paucity of effective treatments for patients with malignant pleural disease. Effusion management and chemotherapy may be the only options depending on the underlying cell type [15, 16]. However, as a significant proportion of patients are unsuitable for chemotherapy due to poor baseline performance score or other comorbidities, there is a requirement for less toxic treatments, which could be used in these frailer patient groups.

Aminobisophosphonates have anti-cancer activity in a number of tumour types through inhibition of farnesyl diphosphate (FPP) synthase (a key element of the mevalonate pathway), resulting in impaired adhesion, migration and proliferation of cancer cells [1]. Additionally, they have direct effects on immune cell activation and inhibit angiogenesis, both of which contribute to their anti-tumour effects. This has led to their widespread use in the treatment and prevention of skeletal related events in cancer and increasing interest in their use as an adjuvant treatment in other cancer types, particularly breast cancer [17].

There is compelling in-vivo and in-vitro evidence to suggest ZA may have beneficial effects in malignant pleural disease. In a mouse model of lung adenocarcinoma affecting the pleural cavity, ZA reduced pleural fluid accumulation and pleural tumour bulk, as well as prolonging survival and limiting cachexia. These effects were associated with reduced new vessel formation and vascular permeability, a reduction in mononuclear cells in the pleural space and a reduction in pro-inflammatory and angiogenic mediators [7].

There is also in vitro evidence that ZA induces apoptosis in mesothelioma cells [4, 6, 18] and favourably affects the host’s immune response to the tumour [5]. Mouse models of mesothelioma also suggest that ZA inhibits tumour growth and prolongs survival [3, 6]. This has led to interest in using ZA in the treatment of malignant pleural disease. There are reports of it being used off licence in mesothelioma [19], however a phase 2 study evaluating its use as an adjuvant treatment for MPE due to non-small cell lung cancer closed prematurely after 3 patients were enrolled due to poor accrual [20].

CT is the current gold standard imaging modality for evaluation of the pleura and the modified RECIST criteria have been developed to stage and monitor treatment response in mesothelioma [21, 22]. Unidimensional measurements from 6 areas of pleural thickening (2 sites at 3 levels) and any enlarged nodes are summed to give the modified RECIST score. The standard definition of a partial response is at least a 30% reduction in this score on 2 occasions 4 weeks apart, for all tumour types [23]. 2 patients in the ZA arm of this study showed a reduction in tumour volume by modified RECIST of >10%, although this does not meet the above criteria for a partial response.

However, these CT results should be interpreted carefully. A number of patients in the trial had small volume disease which may make consistent measurement of pleural tumour thickness more difficult [24]. Recently published data supports our decision to measure disease >5mm and reporting by consensus in this trial aimed to standardise measurements and minimise bias [24]. Additionally, the trial scans were performed only 5 weeks apart, which is a short time interval to detect a treatment response.

DCE-MRI is a novel imaging method, which has recently shown some promise in the evaluation of malignant pleural disease [25, 26]. It monitors the transit of contrast through the tissues and hence characterises the tumour’s vascularity and vascular permeability. This makes it an attractive imaging technique in pleural malignancy, where neovascularisation is felt to be pathogenic and confers a worse prognosis [27]. However, the best technique for reporting DCE-MRI is yet to be established and given the lack of robust, validated methodology, it does not have an established role in routine clinical practice. We did not demonstrate a change in DCE-MRI in response to treatment, which may reflect the analysis methods used and/or the small number of patients with evaluable data.

In animal models, ZA reduces the expression of a number of pro-inflammatory and angiogenic mediators, including VEGF, IL-6 and MCP-1 [7] and an observational study in mesothelioma found falling levels of circulating VEGF to be a good prognostic factor after 8 weeks of chemotherapy [28]. However, despite this and data from other cancer types also suggesting Zoledronic Acid supresses circulating VEGF levels, we failed to show any treatment effect of ZA on the blood biomarkers tested [29, 30]. We may have missed a local effect of the ZA on these inflammatory mediators given the small numbers of patients with indwelling catheters in the trial.

We did not demonstrate any benefit of ZA in terms of symptom control or quality of life compared to placebo in this study. Given the advanced nature of the malignant disease in many of the study patients and the resultant complex, multi-factorial nature of many of their symptoms, this is not entirely surprising in this small cohort. The different tools used to evaluate both overall quality of life and breathlessness all showed consistent results, suggesting that measuring these symptoms with multiple different tools may not add value.

ZA did not appear to be associated with a marked increase in side effects compared to placebo. The side effect profile of ZA is well established due to its extensive use for other indications, but it is reassuring that the treatment appeared to be well tolerated in this patient group [9].

This study was designed as a proof-of-principle pilot study and hence has a number of limitations. We excluded patients receiving other cancer treatments and given the frail nature of this population, recruitment was challenging and the number of included patients is small. In view of this, we were unable to recruit as many patients with indwelling pleural catheters as initially envisaged, limiting our ability to detect a potential signal regarding pleural fluid output and pleural fluid biomarkers. The recruited patients were heterogeneous and the groups poorly balanced for a number of variables at baseline, which may have contributed to the difficulties in identifying a signal for a number of the outcome measures.

There is data from a number of cell types to suggest that the aminobisphosphonates may act synergistically with chemotherapy. A recent study evaluating the potential synergistic effect of intrapleural ZA with systemic Cisplatin chemotherapy in a mouse model of mesothelioma showed combination therapy had greater anti-tumour effects than either agent used alone [18]. We did not evaluate this synergy in the current study, but may be interesting to explore in future trials. We also await with interest the results of an open label trial of ZA in mesothelioma, which is currently recruiting in Birmingham, Alabama [31].

In summary, the results of this small pilot study have not shown conclusive evidence of a treatment effect of Zoledronic Acid in this population. However, given the small number of patients randomised and substantial baseline imbalances between the groups, we cannot exclude a potential beneficial effect. We feel that a further pilot study in a more specific subgroup of patients is warranted to evaluate its effect further. We have learnt important lessons about the feasibility of future studies and this data will help inform trial design and power calculations.

## Supporting Information

S1 CONSORT Checklist CONSORT Checklist. (DOC)

S1 Protocol Trial Protocol. (PDF)

S1 File Patient Information Sheet. (PDF)

S2 File Consent Form. (PDF)

S3 File Supplementary material. (DOCX)

S4 File SOP for DCE MRI analysis. (PDF)

S5 File Statistical analysis plan. (PDF)

## References

[1] Gnant M, Clezardin P. Direct and indirect anticancer activity of bisphosphonates: a brief review of published literature. Cancer Treat Rev 2012;38(5):407–15 10.1016/j.ctrv.2011.09.003 21983264

[2] Clezardin P. Bisphosphonates' antitumor activity: an unravelled side of a multifaceted drug class. Bone 2011;48(1):71–9 10.1016/j.bone.2010.07.016 20655399

[3] Wakchoure S, Merrell MA, Aldrich W, Millender-Swain T, Harris KW, et al Bisphosphonates inhibit the growth of mesothelioma cells in vitro and in vivo. Clin Cancer Res 2006;12(9):2862–8 16675582 10.1158/1078-0432.CCR-05-2766

[4] Kawata E, Ashihara E, Nakagawa Y, Kiuchi T, Ogura M, et al A combination of a DNA-chimera siRNA against PLK-1 and zoledronic acid suppresses the growth of malignant mesothelioma cells in vitro. Cancer Lett 2010;294(2):245–53 10.1016/j.canlet.2010.02.008 20206440

[5] Veltman JD, Lambers ME, van Nimwegen M, Hendriks RW, Hoogsteden HC, et al Zoledronic acid impairs myeloid differentiation to tumour-associated macrophages in mesothelioma. Br J Cancer 2010;103(5):629–41 10.1038/sj.bjc.6605814 20664588 PMC2938257

[6] Okamoto S, Kawamura K, Li Q, Yamanaka M, Yang S, et al Zoledronic Acid Produces Antitumor Effects on Mesothelioma Through Apoptosis and S-Phase Arrest in p53-Independent and Ras prenylation-Independent Manners. J Thorac Oncol 2012;7(5):873–82 10.1097/JTO.0b013e31824c7d43 22481236

[7] Stathopoulos GT, Moschos C, Loutrari H, Kollintza A, Psallidas I, et al Zoledronic acid is effective against experimental malignant pleural effusion. Am J Respir Crit Care Med 2008;178(1):50–9 10.1164/rccm.200710-1513OC 18388351

[8] EORTC. EORTC QLQ-C30 Scoring Manual. 2001. http://www.eortc.be/qol/files/SCManualQLQ-C30.pdf (accessed 19th Sept 2014).

[9] Novartis Pharmaceuticals UK Ltd. Summary of Product Characteristics- zoledronic acid. 2012. http://www.medicines.org.uk/emc/medicine/14062 (accessed 9th August 2012).

[10] Kahan BC, Jairath V, Dore CJ, Morris TP. The risks and rewards of covariate adjustment in randomized trials: an assessment of 12 outcomes from 8 studies. Trials 2014;15:139 10.1186/1745-6215-15-139 24755011 PMC4022337

[11] Kahan BC, Morris TP. Improper analysis of trials randomised using stratified blocks or minimisation. Statistics in Medicine 2012;31(4):328–40 10.1002/sim.4431 22139891

[12] Kahan BC, Morris TP. Reporting and analysis of trials using stratified randomisation in leading medical journals: review and reanalysis. BMJ 2012;345:e5840 10.1136/bmj.e5840 22983531 PMC3444136

[13] White IR, Thompson SG. Adjusting for partially missing baseline measurements in randomized trials. Stat Med 2005;24(7):993–1007 15570623 10.1002/sim.1981

[14] Matthews JN, Altman DG, Campbell MJ, Royston P. Analysis of serial measurements in medical research. BMJ 1990;300(6719):230–5 2106931 10.1136/bmj.300.6719.230 PMC1662068

[15] Vogelzang NJ, Rusthoven JJ, Symanowski J, Denham C, Kaukel E, et al Phase III study of pemetrexed in combination with cisplatin versus cisplatin alone in patients with malignant pleural mesothelioma. J Clin Oncol 2003;21(14):2636–44 12860938 10.1200/JCO.2003.11.136

[16] van Meerbeeck JP, Gaafar R, Manegold C, Van Klaveren RJ, Van Marck EA, et al Randomized phase III study of cisplatin with or without raltitrexed in patients with malignant pleural mesothelioma: an intergroup study of the European Organisation for Research and Treatment of Cancer Lung Cancer Group and the National Cancer Institute of Canada. J Clin Oncol. 2005;23(28):6881–9. 16192580 10.1200/JCO.20005.14.589

[17] Gnant M, Mlineritsch B, Schippinger W, Luschin-Ebengreuth G, Postlberger S, et al Endocrine therapy plus zoledronic acid in premenopausal breast cancer. N Engl J Med 2009;360(7):679–91 10.1056/NEJMoa0806285 19213681

[18] Okamoto S, Jiang Y, Kawamura K, Shingyoji M, Fukamachi T, et al Zoledronic acid produces combinatory anti-tumor effects with cisplatin on mesothelioma by increasing p53 expression levels. PLoS ONE 2013;8(3):e60297 10.1371/journal.pone.0060297 23555949 PMC3610651

[19] Lawson A. Speak Up: Reflections from a Nightmare Patient—Me! Oncology Times 2010;32(12):44

[20] Bushunow P. Zometa Adjuvant Treatment of Malignant Pleural Effusion Due to Non-Small Cell Lung Cancer. http://clinicaltrials.gov/ct2/show/record/NCT01004510?sect=X740156 (accessed 19th August 2014).

[21] Tsao AS, Garland L, Redman M, Kernstine K, Gandara D, et al A practical guide of the Southwest Oncology Group to measure malignant pleural mesothelioma tumors by RECIST and modified RECIST criteria. J Thorac Oncol 2011;6(3):598–601 10.1097/JTO.0b013e318208c83d 21270668 PMC3643692

[22] Byrne MJ, Nowak AK. Modified RECIST criteria for assessment of response in malignant pleural mesothelioma. Ann Oncol 2004;15(2):257–60 14760119 10.1093/annonc/mdh059

[23] Eisenhauer EA, Therasse P, Bogaerts J, Schwartz LH, Sargent D, et al New response evaluation criteria in solid tumours: revised RECIST guideline (version 1.1). Eur J Cancer 2009;45(2):228–47 10.1016/j.ejca.2008.10.026 19097774

[24] Armato SG 3rd, Nowak AK, Francis RJ, Kocherginsky M, Byrne MJ. Observer variability in mesothelioma tumor thickness measurements: defining minimally measurable lesions. J Thorac Oncol 2014;9(8):1187–94 10.1097/JTO.0000000000000211 25157772

[25] Giesel FL, Bischoff H, von Tengg-Kobligk H, Weber MA, Zechmann CM, et al Dynamic contrast-enhanced MRI of malignant pleural mesothelioma: a feasibility study of noninvasive assessment, therapeutic follow-up, and possible predictor of improved outcome. Chest 2006;129(6):1570–6 16778277 10.1378/chest.129.6.1570

[26] Coolen J, De Keyzer F, Nafteux P, De Wever W, Dooms C, et al Malignant pleural disease: diagnosis by using diffusion-weighted and dynamic contrast-enhanced MR imaging—initial experience. Radiology 2012;263(3):884–92 10.1148/radiol.12110872 22535562

[27] Yasumitsu A, Tabata C, Tabata R, Hirayama N, Murakami A, et al Clinical significance of serum vascular endothelial growth factor in malignant pleural mesothelioma. J Thorac Oncol 2010;5(4):479–83 10.1097/JTO.0b013e3181d2f008 20357617

[28] Kao SC, Harvie R, Paturi F, Taylor R, Davey R, et al The predictive role of serum VEGF in an advanced malignant mesothelioma patient cohort treated with thalidomide alone or combined with cisplatin/gemcitabine. Lung Cancer 2012;75(2):248–54 10.1016/j.lungcan.2011.06.007 21757252

[29] Santini D, Vincenzi B, Dicuonzo G, Avvisati G, Massacesi C, et al Zoledronic acid induces significant and long-lasting modifications of circulating angiogenic factors in cancer patients. Clin Cancer Res 2003;9(8):2893–7 12912933

[30] Winter MC, Wilson C, Syddall SP, Cross SS, Evans A, et al Neoadjuvant chemotherapy with or without zoledronic acid in early breast cancer—a randomized biomarker pilot study. Clin Cancer Res 2013;19(10):2755–65 10.1158/1078-0432.CCR-12-3235 23515409

[31] Robert F. Pilot Study of Bisphosphonate Therapy (Zoledronic Acid) in Patients With Malignant Mesothelioma (UAB0901). 2014. http://clinicaltrials.gov/show/NCT01204203 (accessed 10th October 2014).

