# Supplementary material for: A Randomised Controlled Trial of Intravenous Zoledronic Acid in Malignant Pleural Disease: A Proof of Principle Pilot Study
Source: PLoS One. 2015 Mar 17;10(3):e0118569. doi: 10.1371/journal.pone.0118569 (PMC4364455; doi:10.1371/journal.pone.0118569)
Supplement: S1 Protocol — (PDF) [file pone.0118569.s002.pdf]

Bristol Randomised Controlled Trial of Zoledronic Acid in Malignant Pleural Disease (Pilot Study)

A double blind randomised controlled trial examining the effect of intravenous zoledronic acid on pleural tumour progression, pleural fluid production, breathlessness and quality of life in patients with malignant pleural disease .

Chief Investigator:
Dr. Nick Maskell
Version 1.8
Version date: 28/06/2012
R and D number: 2137
REC: 09/H0206/12
EUDRA CT: 2009-009134-32
UKCRN Study ID: 8877
ISRCTN17030426

The Research Team – contact details:

Chief Investigator

Dr. Nick Maskell
Consultant and Senior Lecturer in Respiratory Medicine
North Bristol Lung Centre
North Bristol NHS Trust
Southmead Hospital
Westbury on Trym
Bristol
BS10 5NB
Tel: 0117 323 5838
Mob: 07799726747
Fax: 0117 323 2947
email: nickmaskell@doctors.org.uk

Trial Co-ordinator

Dr. Amelia Dunscombe
Pleural Research Registrar
North Bristol Lung Centre
North Bristol NHS Trust
Southmead Hospital
Westbury on Trym
Bristol
BS10 5NB
Tel: 07810611658
Fax: 0117 323 2947
email: ameliadunscombe@yahoo.co.uk

Other key investigators

1/. Dr Clare Hooper
Pleural Research Registrar
North Bristol Lung Centre
North Bristol NHS Trust
Southmead Hospital
Westbury on Trym
Bristol
BS10 5NB
Tel: 0117 323 5838
Fax: 0117 323 2947
email: clarehooper@doctors.org.uk

2/. Dr. Jeremy Braybrooke
Consultant Oncologist
Bristol Oncology Centre
Horfield Road
Bristol
BS2 8ED
Tel: 0117 928 2418
Fax: 0117 928 2027

email: Jeremy.Braybrooke@UHBristol.nhs.uk

3/. Dr. Mike Darby
Consultant Radiologist
North Bristol NHS Trust
Radiology Dept.
Southmead Hospital
Westbury on Trym
Bristol
BS10 5NB
Tel: 0117 959 5124
Fax: 0117 959 5122
email: Mike.Darby@nbt.nhs.uk

4/. Prof. Iain Lyburn
Professor of Radiology
Colbalt Imaging Centre
Cheltenham General Hospital
Thirlestaine Road
Cheltenham
GL53 7AS
Tel: 01242 535 923
Fax: 01242 535 924
email: iain.lyburn@glos.nhs.uk

5/. David Hall
Consultant Research Physicist
Colbalt Imaging Centre
Cheltenham General Hospital
Thirlestaine Road
Cheltenham
GL53 7AS
Tel: 01242 535 923
Fax: 01242 535 924

Trial Steering Committee Members

Independent chairman: John Harvey
Chief Investigator: Nick Maskell
Trial co-ordinator: Amelia Dunscombe
Trial Statistician: Paul White
Trial Nurse: Anna Morley
Key Investigator: Clare Hooper
Key Investigator: Jeremy Braybrooke
Key Investigator: Mike Darby
Key Investigator: Iain Lyburn
Key Investigator: David Hall
Independent Member: Vidan Masani

Contents

	Page
1.1 Abstract	5
1.2 Lay Summary	5
1.3 Background	8
1.4 References	12
2.1 Research questions	15
2.2 Study protocol flow chart	16
2.3 Setting/Recruitment	17
2.4 Inclusion Criteria	17
2.5 Exclusion Criteria	17
2.6 Standard Care/Baseline tests	17
2.7 Trial interventions	18
2.8 Monitoring of serum calcium, phosphate and magnesium.	20
2.9 Exclusion from continuation with trial.	20
3.1 Outcome measures	21
3.2 Sub group analysis	21
3.3 Statistical analysis	21
3.4 Withdrawal of patients	22
4.1 Drug information	23
4.2 Placebo information	24
4.3 Drug/placebo preparation	24
4.4 Drug/placebo delivery	24
5.1 Randomisation	25
6.1 Biological samples	25
6.2 VEGF-A	25
7.1 Imaging	26
7.2 DCE-MRI	26
7.3 CT thorax scans	26
8.1 Quality of Life measures	27
8.2 Daily dyspnoea VAS	27
9.1 Study timetable	28
10.1 Trial Costs	28
11.1 Infrastructure	28
12.1 SAE/SUSAR reporting	29
13.1 Ethical considerations	29
13.2 Ethical approval	30
13.3 Protocol amendments	29
13.4 Application for ethical approval of protocol amendments	29
Annex A: Daily dyspnoea VAS	31
Annex B: DCE-MRI protocol	32
Annex C: CT thorax protocol	35

1.1 Abstract

Malignant pleural disease is common clinical problem,with effusions occurring in 15% of patients diagnosed with cancer during the course of their disease. They indicate a particularly poor prognosis. Malignant pleural effusions are associated with dyspnoea and recurrent hospital attendances and have a detrimental impact on the quality of life of cancer patients.

The most commonly employed management strategy of thoracocentesis and talc pleurodesis has suboptimal success rates and patients frequently undergo repeated invasive procedures as a result. These strategies seek to drain pleural fluid and attempt to obliterate the pleural space but do not target the principle problem of excess fluid accumulation.

A drug that reduces pleural fluid production would have the potential to improve symptoms in patients with malignant effusions and might have particular utility in the treatment of patients with 'trapped lung' or severe underlying lung disease for whom pleurodesis is relatively contraindicated or indeed for patients with small effusions at presentation where optimum timing of pleurodesis is controversial.

There is a wealth of in vitro and in vivo animal and human evidence to suggest that the amino-bisphosphonate, zoledronic acid (already in common clinical use for skeletal indications) has potent anti-angiogenic and anti-tumour effects. Zoledronic acid has been shown to inhibit growth of mesothelioma cells in mice and reduce pleural fluid accumulation in a maurine model of pleural adenocarcinoma. The addition of ZA to endocrine therapy in breast cancer has recently been associated with highly significant improvements in disease free and relapse free survival.

This pilot study seeks to inform a large multicentre randomised controlled trial examining the effect of zoledronic acid on pleural tumour progression, pleural fluid accumulation, breathlessness and quality of life as compared to placebo in patients with symptomatic malignant pleural effusions and/or thickening of all histological types.

1.2 Lay summary

Cancer commonly spreads to the lining of the lung (the pleura) and may also originate there (mesothelioma) and may be associated with fluid accumulation between the two lung linings. This fluid accumulation is named a 'malignant pleural effusion' and is often associated with unpleasant symptoms of breathlessness and cough as several litres of fluid can form, compressing the lungs. This usually means that the patient's life expectancy is particularly short. We commonly manage this problem by inserting a chest drain to remove all of the fluid which can be done on the ward or during an operation called a thoracoscopy. Draining the fluid is not a

permanent solution as it re-accumulates rapidly in most patients. An attempt to stick the linings of the lung together and take away the space into which fluid can accumulate is usually made. This is called 'pleurodesis'. Sterile talc in the form of a slurry that is injected into the chest drain or a powder which is sprayed over the lung lining during thoracoscopy is most commonly used for pleurodesis. This probably has about a 70% success rate in terms of improvement in pleural fluid volume and associated breathlessness. We cannot use pleurodesis for patients who's lungs fail to fully re-expand after removal of fluid (usually because the lung has been compressed for a long time or there is a lot of tumour over the lung lining). There are ongoing safety concerns regarding the use of talc as it has been (rarely) associated with life threatening side effects. An alternative to pleurodesis and a strategy that is commonly used when pleurodesis is unsuccessful or inappropriate is the placement of a small permanent drain that allows the patient to go home and have fluid drawn off in the community whenever they are breathless. This is usually well tolerated but carries a risk of infection.

These available options do not address the underlying problem of cancer cells in the lining of the lungs causing excessive fluid production.

A drug that acted directly to reduce fluid production has the potential to improve the symptoms of patients for whom drainage and pleurodesis are inappropriate or have proved unsuccessful and possibly even to target malignant pleural effusions early with the aim of avoiding invasive procedures.

Zoledronic acid is a drug that is in common use for patients with cancer that has spread to their bones, for bone thinning (osteoporosis) in post menopausal women and to treat high calcium levels and some other bone disorders. It is given as a drip and can be given as a one off dose or repeatedly at 3-4 weekly intervals. It has been shown that it has effects on cancer cells and particularly their ability to make new blood vessels that may be useful beyond its impact on bone disease. It has been shown to reduce relapse rate in women with breast cancer when added to other usual treatment. Several recent studies have demonstrated exciting results in mouse models of pleural cancers. Zoledronic acid reduces the growth of two kinds of cancer of the lung lining and also appears to reduce pleural fluid production in mice.

This evidence, along with a favourable safety profile in its current context of use, supports a trial of zoledronic acid in malignant pleural disease. There are many unanswered questions that warrant study via clinical trials. Although the drug certainly has an anti-cancer effect, we do not know whether the impact seen on pleural tumours and fluid production in mice will translate to humans. Safe dosing of zoledronic acid has been established but optimum dosing

for anti cancer activity in the pleural space (between the lung linings) in humans is not known.
This study seeks to examine whether zoledronic acid at its currently licensed dose reduces the progression of pleural tumours, accumulation of pleural fluid and improves symptoms in patients with malignant pleural disease. We intend to perform a small pilot study in the first instance.

1.3 Background

Malignant pleural disease is a common clinical condition with malignant pleural effusion (MPE) affecting 3000 people per million of the population per year. MPE is associated with significant health care costs and has a negative impact on cancer patients' quality of life.

Up to 50% of patients with breast cancer and 25% patients with primary lung cancer will develop a pleural effusion during the course of their disease. While lung and breast cancer are the most common causes of MPE, it also occurs in over 95% of patients with malignant pleural mesothelioma and is common in lymphoma and many other primary adenocarcinomas. Malignant pleural effusions indicate advanced and disseminated disease and are associated with a poor prognosis with life expectancy varying from 3-12 months depending on primary tumour type. Effusions associated with primary lung cancer predict a particularly short life expectancy.

The mechanism underlying development of MPE has not been fully elucidated. Metastatic spread to the visceral pleura occurs haematogenously with secondary seeding of the parietal pleura[1].Less frequently, tumour reaches the pleural space by direct invasion from adjacent structures. While both increased pleural fluid production and reduction in drainage due to lymphatic blockage are responsible for fluid accumulation, the former probably plays the more important role[2,3]. Malignant cells in the pleural space, interact with resident mesothelial cells and circulating inflammatory cells to induce an environment of increased vascular permeability and neovascularisation via the production of cytokines[4]. A key component of this process and arguably the most thoroughly studied of the relevant cytokines is VEGF (vascular endothelial growth factor. ie VGEF-A). VEGF induces increased vascular permeability and vasodilatation, stimulates cell proliferation and migration and inhibits apoptosis. Its role in MPE formation is in the induction of increased permeability of the vascular and mesothelial barriers of the parietal pleura to plasma and cells and as a growth factor to neoplastic cells[5].It is present at consistently higher levels in exudate than transudate effusions and within the exudates is higher in those of a malignant aetiology[6,7].VEGF levels in malignant effusions are up to ten times of those in matched serum, indicating that local production, rather than diffusion from the circulation is most important[8]. Resident mesothelial cells express VEGF receptors and produce VEGF and are likely to be the main source of the cytokine in non-malignant effusions. VEGF is an important angiogenic growth factor for neoplasms and is heavily expressed by most malignant cells types which are likely to be the prominent source in malignant effusions[9]. Serum VEGF levels have been shown to correlate inversely with patient survival in mesothelioma and predict response to chemotherapy in small cell lung cancer [10,11].

Malignant pleural effusions frequently occupy more than half of a hemithorax and present with progressive exertional dyspnoea and sometimes cough. Established strategies for the management of MPE are associated with several problems and limitations. Without pleurodesis symptomatic pleural fluid reaccumulation occurs following initial drainage in 70-90% of patients [12]. This is often associated with a deterioration in quality of life and repeated hospital attendances. Chemical pleurodesis with talc slurry or poudrage is the most effective and most commonly used pleurodesis strategy in malignant effusions worldwide. Pleurodesis with talc has been shown to have between a 68% and 94% success rate (partial and complete success combined) in clinical trials [13-15]. In routine clinical practice, success rates with talc slurry are often observed to be at the lower end of the range demonstrated in the clinical trial environment. Repeat talc pleurodesis in those with a failed first attempt has been shown to have a 50% success rate [16]. An alternative and increasingly exploited strategy in this situation is the placement of an indwelling pleural catheter which allows the patient to undergo regular thoracocentesis in the community at a frequency guided by their dyspnoea. Placement of an indwelling pleural catheter has also been associated with up to a 58% spontaneous pleurodesis rate[17].

Talc pleurodesis has been linked with the life threatening side effect of ARDS with an incidence of 0-9%. ARDS appears to be more common when small particle (<20μm) or mixed particle size talc is used due to its potential for systemic distribution. Additionally, talc pleurodesis may impair gas exchange and should be avoided in patients with underlying lung disease associated with hypoxia[18].In light of the ongoing re-examination of its safety by the Medicines and Healthcare Products Regulatory Agency, in the UK the use of talc for pleurodesis is currently unlicensed and prescribed on a named patient basis.

Talc pleurodesis is contraindicated and usually unsuccessful when there is incomplete lung re-expansion following ward based tube drainage or drainage at thoracoscopy. This phenomenon of 'trapped lung' is common following drainage of a large pleural effusion that has been present for some time and in malignant pleural mesothelioma or other pleural malignancy where the visceral pleura is encased by tumour. Indwelling pleural catheter placement is now frequently employed in cases of trapped lung. Recognised complications include pleural space infection and malignant track invasion (particularly in malignant pleural mesothelioma).

The strategies described address fluid removal and obliteration of the pleural space but not the primary problem of excess pleural fluid production. The suboptimal success rate and potential complications associated with chemical pleurodesis motivate a search for novel and superior treatments with the

potential to avoid invasive interventions and reduce patient dyspnoea and hospital visits.

Agents that interact with angiogenic growth factors are of particular interest and the monoclonal antibody to VEGF; bevicuzimab has been used with some success in a small number of patients with benign effusions[19].

Zoledronic acid is an amino-bisphosphonate which is licenced in the UK for the treatment of hypercalcaemia of malignancy, pagets disease, osteoporosis and the prevention of pathological fractures in advanced malignancy. In these contexts, it has been shown to have a favourable safety profile (see section 4.1). A series of recent animal and in vitro and in vivo human studies have demonstrated anti- angiogenic and anti-tumour properties of the drug. Amino-bisphosphonates impair the survival, invasiveness and angiogenesis of malignant cells via potent inhibition of farnesyl diphosphate synthase in the mevalonate pathway and of prenylation of small G-proteins, a mechanism also responsible for their osteoclast mediated antiresorptive effect on bone. Additionally, they have immunostimulatory properties, promoting the the expansion of tumoricidal T cells[20].Zoledronic acid displays activity against many cancer cell types in vitro and in vivo and has been shown to reduce circulating VEGF when administered to patients with cancer[21,22] . A large randomised clinical trial has recently demonstrated a highly significant improvement in disease and relapse free survival in premenopausal women with hormone responsive breast cancer when zoledronic acid is added to endocrine therapy [23]. This translation of a wealth of in vitro and animal evidence for a drug that is already in common use for skeletal indications, into demonstrable clinical benefit has stimulated interest in other cancer settings.

Could zoledronic acid also have a role in the management of malignant pleural disease? Zoledronic acid inhibits tumour growth and prolongs survival in vivo in mice with mesothelioma[24]. A recent study demonstrated a reduction in pleural fluid accumulation, pleural tumour dissemination and cachexia and significantly improved survival with ZA vs placebo in a maurine model of pleural adenocarcinoma with associated reduction in tumour expression of angiogenic and inflammatory mediators including VGEF, MIP-2 and IL-6[25]. In this elegant animal study Stathopoulos et al used a clinically meaningful dose as the 100μg/kg used is equivalent to the human dose of 4 mg 3-4 weekly[26].

The bioavailability of zoledronic acid to the pleura is not known. Bisphosphonates are rapidly cleared from the circulation following IV administration and are absorbed and retained in bone matrix for prolonged periods[27]. The bone scan agent $^{99}TC^{m}$ diphosphonate is structurally similar to bisphosphonates and is well documented to accumulate in malignant pleural effusions

suggesting that bioavailability to a pleural site of action may be favourable[28].

We plan initially to perform a small pilot study in order to establish a sample size calculation for a randomised controlled trial examining the benefit of adding zoledronic acid to usual clinical management for patients with malignant pleural disease of all histological types.

References

1/. Rodriguez-Pandero F, Borderas Naranjo F, Lopez- Mejias J. Pleural metastatic tumours and effusions. Frequency and pathogenic mechanisms in post-mortem series. Eur Resp J. 1989; 2: 366-9.

2/. Meyer PC. Metastatic carcinoma of the pleura. Thorax 1966; 21: 437-43.

3/. Light RW, Hamm H: Malignant pleural effusion: would the real cause please stand up? Eur Respir J 1997, 10: 1701-1702.

4/. Mutsaers SE, Wilkosz S. Structure and function of mesothelial cells. Cancer Treat Res 2007; 134: 1-19.

5/. Mohammed KA,Nasreen N, Hardwick J, et al. Bacterial induction of pleural mesothelial monolayer barrier dysfunction. Am J Physiol Lung Cell Mol Physiol. 2001, 281: L119- L125.

6/. Thickett DR, Armstrong L, Millar AB: Vascular endothelial growth factor (VGEF) in inflammatory and malignant pleural effusions. Thorax 1999, 54:707-710.

7/. Yanagawa H, Takeuchi E, Suzuki Y et al. Vascular endothelial growth factor in malignant pleural effusion associated with lung cancer. Cancer Immunol Immunother 1999, 48: 396-400.

8/. Kraft A, Weindel K, Ochs A et al: vascular endothelial growth factor in the sera and effusions of patients with malignant and non-malignant disease. Cancer 1999, 85:178-187.

9/. Grove CS, Lee YCG: Vascular endothelial growth factor: the key mediator in pleural effusion formation. Current Opinion in Pulmonary Medicine, 8(4): 294-301.

10/. Strizzi L, Catalano A, Vianale G, et al: Vascular endothelial growth factor is an autocrine growth factor in human mesothelioma. J Pathol 2001, 193: 468-475.

11/. Salven P, Ruotsalainen T, Mattson K et al: High pre-treatment serum level of vascular endothelial growth factor (VEGF) is associated with poor outcome in small-cell lung cancer. Int J Cancer 1998, 79:144-146.

12/. Anderson CB, Philpott GW, Ferguson TB. The treatment of malignant pleural effusions. Cancer 1974. 33: 916-22.

13/. Dresler CM, Olak J, Hendon JE, et al. Phase III intergroup study of talc poudrage vs talc slurry sclerosis for malignant pleural effusion. Chest 2005; 127: 909-15.

14/. Adler RH, Sayek I. Treatment of malignant pleural effusion: a method using tube thoracostomy and talc. Ann Thorac Surg 1976; 22: 8-15.

15/. Noppen M, Degreve J, Mignolet M, Vincken W. A prospective, randomised study comparing the efficacy of talc slurry and bleomycin in the treatment of malignant pleural effusions. Acta Clin Belg 1997; 52:258-62.

16/. Dikensoy O, Light RW. Alternative widely available inexpensive agents for pleurodesis. Curr Opin Pulm Med 2005; 11: 340-4.

17/. Saffran L, Ost DE, Fein AM, Schiff MJ. Outpatient pleurodesis of malignant pleural effusions using a small bore pigtail catheter. Chest 2000; 118: 417-21.

18/. Janssen JP, Collier G, Astoul P et al. Safety of pleurodesis with talc poudrage in malignant pleural effusion: a prospective cohort study. Lancet 2007; 369: 1535- 1539.

19/. Hoyer RJ, Leung N, Witzig TE, Lacy MQ. Treatment of diuretic refractory pleural effusions with bevicuzimab in four patients with primary systemic amyloidosis. Am J Hematol 2007; 82: 409-413.

20/. Miyagawa F, Tanaka Y, Yamashita S, Minato N. Essential requirement of antigen presentation by monocyte lineage cells for the activation of primary γδ T cells by aminobisphosphonate antigen. J immunol 2001; 166: 5508.

21/. Yuasa T, Kimura S, Ashihara E, Habuchi T, Maekawa T. Zoledronic acid: a multiplicity of anti-cancer action. Curr Med Chem 2007; 14: 2126-2135.

22/. Santini D, Schiavon G, Angeletti S, Vincenzi B et al. Last generation of amino-bisphosphonates (N-BPs) and cancer angiogenesis: a new role for these drugs? Recent Patients Anticancer Drug Discov 2006; 1: 383: 396.

23/. Gnant M, Mlineritsch B, Schippinger W, Luschin-Ebengreuth G et al. Adjuvant ovarian suppression combined with tamoxifen or anastrozole, alone or in combination with zoledronic acid, in premenopausal women with hormone-responsive, stage I and II breast cancer: First efficacy results from ABCSG -12. Journal of Clinical Oncology 2008. 26, 15S (May 20 supplement).

24/. Wakchoure S, Merrell MA, Aldrich W, Millender-Swain T. Bisphosphonates inhibit the growth of mesothelioma cells in vitro and in vivo. Clin Cancer Res. 2006; 12(9): 2862-2868.

25/. Strathopoulos GT, Moschos C, Loutrari H, Kollintza A et al. Zoledronic acid is effective against experimental malignant pleural effusion. Am J Respir Crit Care Med 2008; 178:50-59.

26/. Winter MC, Holen I, Coleman RE. Exploring the anti-tumour activity of bisphosphonates in early breast cancer. Cancer Treatment Reviews 2008;34:453-475.

27/. Fleisch H. Bisphosphonates. Pharmacology and use in the treatment of tumour-induced hypercalcaemic and metastatic bone disease. Drugs 1991; 42:919-44.

28/. Sandler ED, Hattner RS, Parisi MT, Miller TR. Clinical utility of bone scan features of pleural effusion: sensitivity and specificity for malignancy based on pleural fluid cytopathology. J Nucl Med 1994; 35:429-431.

29/. Wright JG, Boddy AV, Highley et al., Estimation of glomerular filtration rate in cancer patients. Br J Cancer 2001; 84(4): 452-9.

2.1 Research questions

In patients with malignant pleural disease:

1/. Is zoledronic acid treatment associated with a reduction in pleural tumour volume, blood flow and angiogenesis when compared to placebo as measured by serial dynamic contrast enhanced MRI and pleural fluid VEGF-A?
2/. Does zoledronic acid reduce dyspnoea and improve quality of life compared to placebo?
3/. Does zoledronic acid reduce pleural tumour progression as measured by CT with volumetric reporting?

Additionally, in patients with a malignant pleural effusion and indwelling pleural catheter:
1/. Does zoledronic acid reduce pleural fluid accumulation compared to placebo?
2/. What is the pleural fluid concentration of zoledronic acid at 7, 14 and 21 days following IV administration of a 4mg dose.

2.2 Study protocol flow chart

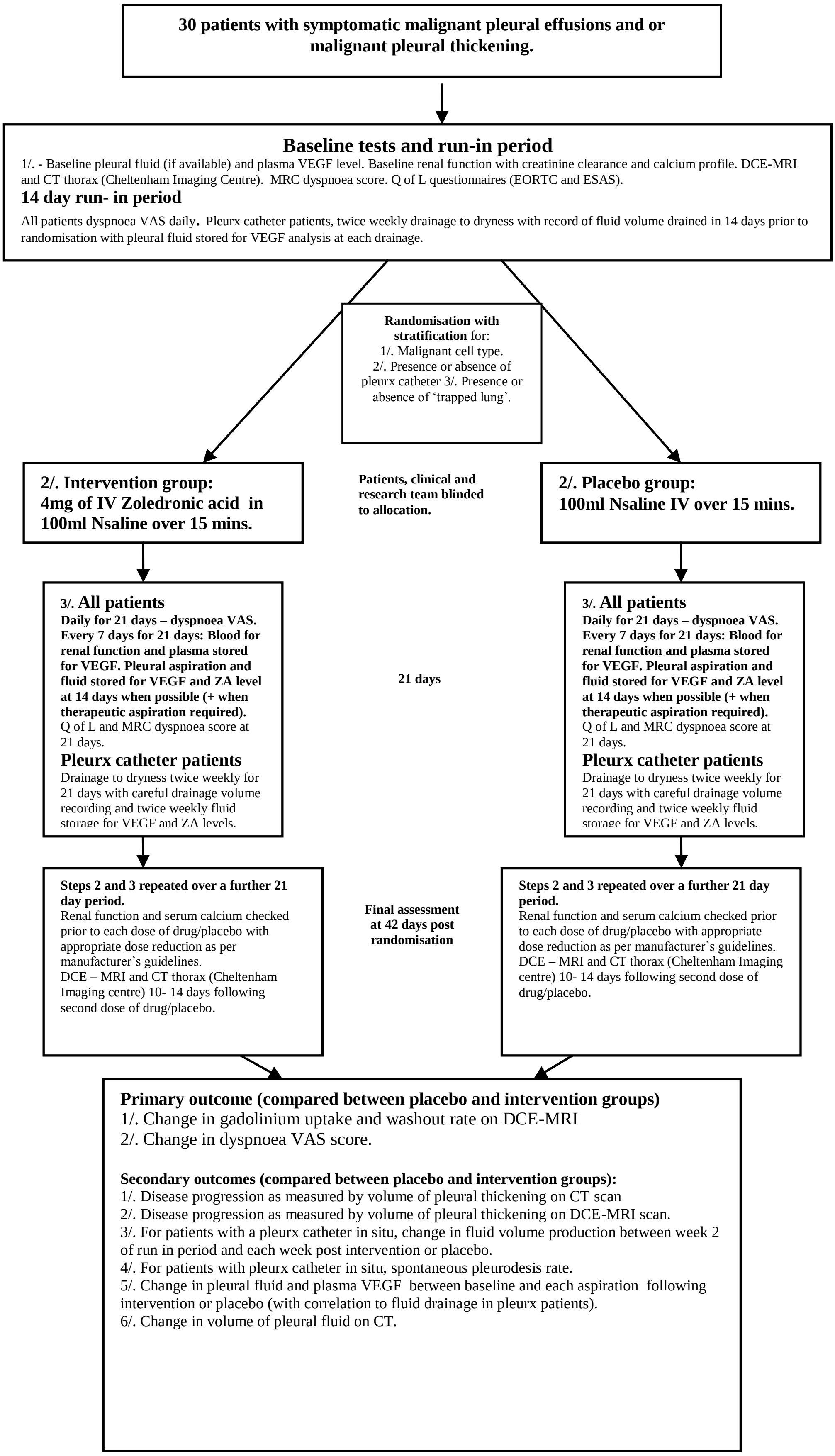

2.3 Setting/recruitment

Patients for the pilot study will be recruited from the respiratory department and oncology clinics of North Bristol NHS trust and The Bristol Oncology Centre. If recruitment targets are not met, the trial steering committee will consider opening further centres (already identified). Consecutive patients fulfilling the inclusion criteria will be invited to participate and consented at their base hospital. All further trial visits, routine clinical care and data collection will take place within the North Bristol lung centre.

2.4 Inclusion criteria

1/. Malignant pleural thickening with or without pleural effusion
a. with malignant fluid cytology or
b. with malignant pleural biopsy histology or
c. in the context of clinically proven cancer elsewhere with no alternative cause found for the pleural thickening or effusion
and
2/. Age > 18 years

2.5 Exclusion criteria

1/. Chemical or surgical pleurodesis in the preceding 30 days.
2/. IV bisphosphonate within the past 3 months or ongoing therapy.
3/. Ongoing dental disease requiring intervention.
4/. Significant renal failure (calculated creatinine clearance of <40ml/min)
5/. Hypocalcaemia at randomisation.
6/. Inability to give informed consent.
7/. Pregnancy or lactation.
8/. Known allergy to bisphosphonates or exipients in the intervention preparation.
9/. Life expectancy < 4 months.
10/. Current or planned chemotherapy (However patients receiving the oral chemotherapy agent, tarceva who have been on it for more than 3 months can be included).
11/. Hormone manipulation therapy initiated in the month before trial entry (however patients receiving long term hormone manipulation can be included).
12/. Haematological malignancy.
13/. Age < 18 years (no upper age limit).
14/. Severe visual impairment.

2.6 Standard care

It is anticipated that approximately 50% of the patients recruited to the trial will have a Pleurx catheter in situ for clinical management of their pleural effusion.

The clinical indications and procedure for Pleurx placement will not be altered by patient participation in the trial.
Within the North Bristol Lung Centre, Pleurx catheters are placed by experienced operators under sterile conditions on the weekly thoracoscopy list. Some patients from The Bristol Oncology Centre may have their pleurx catheters inserted by the thoracic surgeons at the Bristol Royal Infirmary. Pleural catheter care is supervised both in hospital and the community by experienced specialist nursing staff who are based in the lung unit.
Patients would usually undergo pleural fluid drainage and follow up both at clinic appointments and at home as indicated by breathlessness.
For a significant proportion of patients (up to 58% in some series) spontaneous pleurodesis occurs after placement of a catheter. In these cases, catheters are removed under sterile conditions in the lung centre. For other patients, pleural fluid continues to be drained and catheters remain in situ indefinitely providing there is no evidence of infection and they continue to improve patient comfort.
The standard care of other patients with malignant pleural disease includes regular specialist review and aspiration or drainage of pleural fluid when indicated by breathlessness. The frequency of review, pleural procedures and imaging varies between patients.

2.7 Trial Interventions

Baseline tests and run in period – 14 days

All patients

Patients will be screened.
Written consent will be taken.
Blood will be taken for full blood count, calculated creatinine clearance, calcium, phosphate and magnesium and plasma and serum stored for future VEGF-A and cytokine analysis.
The creatinine clearance will be calculated using the Wright equation[29]
Pleural fluid will be taken at Pleurx catheter drainage and stored for future VEGF-A and cytokine analysis.
Patients will complete a visual analogue scale of breathlessness(see annex A) at the initial visit and then daily for 14 days.
The MRC dyspnoea scale and quality of life questionnaires (the Edmonton Symptom Assessment Scale (ESAS) and the EORTC QLQ – C30) will be completed.
Patients will undergo a specialist dental check-up during the run-in period at the Department of Oral and Maxillofacial Surgery at Southmead Hospital.
Patients will undergo a CT scan and DCE-MRI scan in a single appointment at the Cheltenham Imaging Centre. Under exceptional

circumstances, occasional scans may be performed at Southmead Hospital.
Providing that no exclusion criteria have been revealed by the baseline blood results or dental check-up, they will be randomised to intervention or placebo on day 7.

If a potential dental problem is identified at the dental assessment, which would preclude treatment with zoledronic acid, patients will be offered the choice of withdrawing from the study, or suspending trial participation until the dental problem has been resolved. In patients who undergo dental treatment during the run in period, a re-assessment by the dentist at the department of oral and maxillofacial surgery at Southmead Hospital will be arranged at least 3 weeks after completion of the dental treatment. At this appointment, if the clinician is satisfied that the dentition is now satisfactory, trial participation can be resumed. When patients re-enter the study, baseline bloods will be taken to recheck the renal function and an up to date creatinine clearance will be calculated. They must complete at least 1 week of VAS scores and pleural fluid collection (if a pleurx is in situ) prior to the first dose of IMP being administrated.

<u>Patients with a Pleurx catheter in situ – drainage protocol.</u>
Patients will be consented and enter the run-in period a minimum of 14 days after catheter placement to allow complete wound healing. Their catheters will be drained to dryness twice a week by the pleural trial nurse or a specialist lung cancer nurse from North Bristol NHS Trust trained in the use of indwelling pleural catheters for the duration of the trial and the volume drained recorded. In most cases, one drainage will be performed in hospital and one in the patient’s home each week.
Drainage to dryness equates to drainage of the maximum possible pleural fluid volume in the absence of patient discomfort. A pleural fluid sample will be frozen for VEGF-A assay and zoledronic acid levels at baseline and every fluid drainage thereafter.

Post randomisation interventions
Serum creatinine, full blood count and corrected calcium, magnesium and phosphate will be measured and checked prior to intervention and blood stored for VEGF-A.
Quality of life questionnaires and MRC dyspnoea score will be repeated.
Patient randomisation will be co-ordinated via an online randomisation service (sealed envelope) with the randomisation list provided directly to pharmacy.

The dose of zometa will depend on the estimated creatinine clearance, as calculated using the Wright equation:

Estimated creatinine clearance (ml/min) as calculated by the Wright Equation	Dose of zoledronic acid (mg)
≥60	4
≥50- <60	3.5
≥ 40-<50	3.3

Zometa (zoledronic acid) in 100ml 0.9% sodium chloride or placebo in the form of 100ml 0.9% sodium chloride will be prepared and delivered to the respiratory department procedure room.

Patients, clinical and research staff will be blind to allocation.
Patients will be advised to attend for intervention/ placebo well hydrated.
A single dose of drug or placebo will be delivered intravenously over 15 minutes.
Patients without evidence of hypercalcaemia will be prescribed calcium 500mg and vitamin D 400 IU once a day for the duration of the trial. In those patients with hypercalcaemia, calcium and vitamin D will not be commenced but the calcium will be monitored regularly during the trial and it will be started should the calcium drop below the normal range.

Patients will continue to complete a daily dyspnoea VAS and will be seen by the trial team every 7 days when blood will be taken for calculated creatinine clearance. If possible, samples will also be stored for future VEGF-A analysis.
Pleural fluid will be stored for VEGF-A and Zoledronic acid levels at each drainage post intervention or placebo for Pleurx patients and whenever a pleural aspiration is clinically indicated for non-pleurx patients.
Quality of life questionnaires and MRC dyspnoea score will be repeated at the end of the 21 day period.

A thoracic ultrasound will be performed at baseline and every 21 days, measuring pleural fluid depth at 3 sites over the affected hemithorax.

The above intervention or placebo and subsequent drainage, and monitoring process will be repeated over a further 21 day period. Serum creatinine, corrected calcium, magnesium and phosphate will be measured before each dose of intervention or placebo.

DCE-MRI scan and CT scan (Cheltenham Imaging Centre) will be repeated 10-14 days following the 2nd dose of drug or placebo. Under exceptional circumstances, occasional scans may be performed at Southmead Hospital as per the same protocol that is used at Cheltenham. Due to the funding structure of the trial, this is only possible for very few patients.

Importantly, patients requiring drainage for breathlessness between weekly visits will receive this care in the community or the hospital respiratory department under the direct supervision of the specialist pleural nurse or a lung cancer nurse specialist from North Bristol NHS Trust, with the additional fluid volume drained recorded and added to that 7 day total.

2.8 Monitoring of serum calcium, phosphate and magnesium

A serum sample will be sent for corrected calcium, magnesium and phosphate in the 24 hours prior to each infusion of ZA or placebo.

Local laboratory normal ranges: Corrected calcium 2.10 – 2.60 mmol/l
Phosphate 0.85 – 1.40 mmol/l
Magnesium 0.7 – 1.0 mmol/L

Hypocalcaemia at randomisation is an exclusion criteria.
A fall in corrected calcium, phosphate or magnesium to below the normal range will be corrected with oral calcium, magnesium and phosphate supplementation and the next dose of intervention delayed for up to 14 days. If salts remain low after 14 days of supplementation, no further ZA or placebo will be administered and the patient included in an intention to treat analysis.
Patients with a history of hypocalcaemia, hypophosphataemia or hypomagnesaemia during the trial will undergo weekly blood tests to ensure that salts remain within the normal range.

2.9 Criteria for exclusion from continuation with trial following randomisation

1/. Serum creatinine increases by >44 μmol/l.
2/. Development of dental problems requiring intervention.
3/. Persistent biochemical dehydration.
4/. New anaemia requiring transfusion, unrelated to clear evidence of haemorrhage (ie suspected drug effect).

3.1 Outcome measures

Primary outcome

1/. Change in gadolinium uptake and washout rate on DCE-MRI
2/. Change in dyspnoea VAS score.

Secondary outcomes

1/. Disease progression as measured by volume of pleural thickening on CT scan
2/. Disease progression as measured by volume of pleural thickening on DCE-MRI scan.
3/. For patients with a pleurx catheter in situ, change in fluid volume production between week 2 of run in period and each week post intervention or placebo.
4/. For patients with pleurx catheter in situ, spontaneous pleurodesis rate.
5/. Change in pleural fluid and plasma VEGF-A between baseline and each aspiration following intervention or placebo (with correlation to fluid drainage in pleurx patients).
6/. Change in volume of pleural fluid on CT.

We will also collect pleural fluid bioavailability data via drug concentration assays.

3.2 Sub group analysis

1/. Chemotherapy or endocrine therapy initiated during trial vs. no systemic therapy initiated.
2/. Malignant cell type.
3/. Presence or absence of 'trapped lung' (defined as incomplete lung re-expansion on plain chest x-ray following initial drainage to dryness at thoracoscopy, ward based intercostal drain or indwelling catheter placement).
4/. Pleurx catheter in situ.

3.3 Statistical analysis

All data will be screened and assessed for unusual outlying or influential values so that robust conclusions may ultimately be drawn from the study.
The control of breathlessness will be measured using the average daily visual analogue scale (VAS) defining breathlessness. This methodology mirrors the technique used to quantify chronic pain in pain trials and in previous pleural effusion trials. VAS has been is shown to be robust and reproducible. A comparison between the two groups VAS scores will be undertaken using standard independent samples test (independent samples t-test or the Mann Whitney test if the shape of the distribution indicates severe non-normality. Measures of effect size (Cohen's d) will be derived using the pilot study data to inform the power calculations for the main

study.
The delta change in gadolinium uptake between each patients DCE-MRI scans will be calculated. A comparison between the two groups will then be undertaken using standard independent samples test (independent samples t-test or the Mann Whitney test if the shape of the distribution indicates severe non-normality).
In a sub-group analysis, the proportion of patients, enrolled with pleurx catheters, achieving spontaneous pleurodesis in each group, will be assessed using the chi-square test of association . The odds ratio from this cross-tabulation will provide an estimate of the effect size for group differences on pleurodesing.
A full analysis plan, will be drawn up by the steering committee, before trial recruitment finishes and the unblinding of the raw data.

3.4 Withdrawal of patients

An intention to treat analysis will be performed.
Data collected to the point at which a patient withdraws from the study will be included in final analysis unless consent for this is withdrawn.

4.1 Drug information

The trial will use zoledronic acid in the form of Zometa 4mg/5ml concentrate for solution.
Zometa is produced by Novartis Pharmaceuticals UK Ltd.
Marketing Authorisation Number: EU/1/01/176/004-006.

Licence:
Zoledronic acid is an amino-bisphosphonate that is licensed and in common use for the treatment of malignant hypercalcaemia, bone metastases from solid tumours, osteolytic lesions from multiple myeloma, post menopausal osteoporosis and pagets disease.

Pharmacokinetics:
Oral bioavailability is poor and it is therefore administered intravenously. ZA has a low affinity for the cellular constituents of blood and is rapidly cleared from the circulation with preferential deposition in calcified tissues.
Zoledronic acid does not appear to undergo metabolism and is ultimately excreted renally with 39% of the administered drug present unchanged in urine over 24 hours. The half life of the drug is 146 hours.

Dose and delivery:

The licensed dose of zometa will depend on the estimated creatinine clearance:

Estimated creatinine clearance (ml/min)	Dose of zoledronic acid (mg)
≥60	4
≥50- <60	3.5
≥ 40-<50	3.3

The dose is repeated at 3-4 weekly intervals for some indications.

It is recommended that serum corrected calcium is checked prior to each dose and that calcium and vitamin D supplements are given throughout the treatment course for all patients without current or previous hypercalcaemia.

Renal failure:
There is a 2% risk of nephrotoxicity which has been largely confined to patients with severe renal failure, hypercalcaemia and dehydration (patients excluded from this trial). To further reduce this risk, it is advised that the dose is reduced in the presence of mild to moderate renal failure.
If creatinine increases 44μmol/l above baseline, it is advised that the drug is omitted until serum creatinine falls to within 10% of baseline when, in clinical practice it is resumed.
For the purposes of this pilot study, patients with calculated creatinine clearance of ≥50-<60 ml/min at randomisation will receive a reduced dose of 3.5mg zoledronic acid. If the estimated creatinine clearance is 40-<50 ml/min, they will receive 3.3mg of zoledronic acid. Patients with creatinine clearance of < 40ml/min are excluded from the trial. If serum creatinine increases by > 44μmol/l above baseline, no further intervention or placebo will be administered and the patient's data included in the intention to treat analysis.
Patients will be advised to drink plenty of fluids prior to attending for each dose of drug/placebo.

Side effect profile:
Zoledronic acid is well tolerated but has been associated with infrequent mild side effects.
Immediate (hours to days after administration) – fever and flu' like symptoms(7%), rash (1%), arthralgia/myalgia (1%), pain(1%).
Early (days to weeks)- asymptomatic hypocalcaemia (6%), hypophosphataemia (4%), (nausea and vomiting (1%), change in taste and thirst (1%), pain (1%), fatigue (1%), myelosupression (1%).

Late (months to years). A serious side effect of jaw osteonecrosis has been reported extremely rarely. This occurs largely in patients undergoing invasive dental work during treatment. Patients with significant dental disease are excluded from this study and all patients will undergo a dental examination prior to drug administration.
Another extremely rare side effect of ZA is an increased risk of atypical femoral fractures. This is usually seen in patients receiving zoledronic acid on a long term basis for osteoporosis and hence is unlikely to be relevant in this study as patients will only receive 2 doses.

4.2 Placebo information

The placebo will take the form of 100ml 0.9% sodium chloride.
Manufactured by Baxter Healthcare Ltd.
Manufacturing authorisation number for Baxter saline 00116/ 0334
Product licence: 0116/0334
The product licence is for:
-Treatment of isotonic extracellular dehydration.
-Treatment of sodium depletion
- Vehicle or diluent of compatible drugs for parenteral administration.
No adverse effects relating to the placebo itself are anticipated.

4.3 Drug/placebo preparation

Drug and placebo will be prepared and labelled in the clinical trials department of North Bristol NHS trust, Southmead site pharmacy.

4.4 Drug/placebo delivery

The intervention will be administered following review of renal function and serum calcium, magnesium and phosphate levels by a physician from the clinical trial team.
An intravenous cannula will be placed and the drug administered by the pleural research nurse in the respiratory department procedure room.
The patient will be observed for 30 minutes following drug delivery and the cannula removed.

5.1 Randomisation

Patients will be block randomised with stratification for:
1/. Malignant cell type.
2/. Presence or absence of Pleurx catheter.
3/. Presence or absence of ‘trapped lung’.

Randomisation will be achieved by an internet randomisation service to ensure complete allocation concealment from the trial team.

6.1 Biological samples

Every 7 days, 10 ml – 20ml blood will be taken. Prior to drug administration and at screening, 5ml in a serum gel tube for standard renal and bone profile, 2.5ml in an EDTA tube for full blood count. Every 7 days following the run through period, 2.5ml in an EDTA tube for plasma VEGF-A and 5ml in a serum gel tube for cytokine and drug concentration will also be taken when possible.
When obtained, 10ml pleural fluid will be stored in 1X EDTA and 2X serum gel tubes for future VEGF-A, cytokine and drug concentration analysis.
Pleural fluid for drug assay will be frozen at -70oc without further processing.
Blood and pleural fluid for VEGF-A and cytokines will be centrifuged as per manufactures guidelines, 30 minutes after collection with resultant 1ml plasma, serum and pleural fluid aliquots frozen at -70oc.
Frozen samples will be stored in a linked anonymised form in a -70 freezer with a locking system only accessible to the clinical trial team.

6.2 VGEF-A

Ultimately the Quantiglo ELISA assay from R and D systems will be used for VEGF quantification. This assay is not yet available to us and the samples will therefore be prepared and frozen according to the assay manufacturers instructions.
We will perform a validation study for pleural fluid analysis with the Quantiglo ELISA using the trial samples. The ELISA is already validated for use with plasma.

7.1 Imaging

Patients will undergo a CT scan and DCE-MRI scan at the Cheltenham Imaging Centre at baseline and 10-14 days following the second and final dose of trial drug or placebo. Under exceptional circumstances, very occasional scans may be performed at Southmead hospital, but due to the funding of the trial, this will only be possible for a few patients.

7.2 DCE-MRI scans

DCE-MRI is a non-invasive radiological technique involving the acquisition of serial MRI images following the intravenous injection of gadolinium. The intensity of gadolinium uptake and speed of washout from tissues has been used to determine malignant disease extent and activity as these measures are markers of tumour microvascular circulation and angiogenesis. A radiological assessment of pleural tumour vascularity is particularly relevant to the proposed mechanism of action of zoledronic acid in malignancy and gadolinium uptake and washout rates may correlate to pleural fluid VEGF-A levels. DCE-MRI used in the monitoring of several solid organ tumours and has been shown to have promise in predicting prognosis and monitoring response to chemotherapy in malignant pleural mesothelioma.
DCE-MRI does not involve any radiation exposure.
Scans will be reported by Prof. Iain Lyburn.
See annex B for the DCE- MRI scan protocol.

7.3 CT scans

See annex C for CT thorax protocol.

8.1 Quality of Life measures

2 questionnaires will be used at baseline, prior to the first intervention, and at the end of each 21 day period.
The EORTC QLQ-C30 is a 30 item generic questionnaire. It was designed for use in chemotherapy trials and has been validated (against robust measures such as WHO performance status, 6 minute walk test and tumour stage) in a palliative setting in patients with lung cancer or mesothelioma and in patients with mesothelioma receiving chemotherapy with gemcitabine and cisplatin[22, 23]
Edmonton Symptom Assessment Scale (ESAS) is a ten item visual analogue scale validated in a clinical setting for the assessment of symptoms and quality of life in cancer patients receiving chemotherapy and patients receiving palliative care[25].

Both questionnaires include a breathlessness scale and can be completed within 10 minutes in combination.

8.2 Daily dyspnoea visual analogue scale.

The VAS will be provided to patients in booklets which will be returned at each visit (total of 8 booklets per patient).
See annex A.

9.1 Study timetable

It is anticipated that recruitment to the amended study will commence in June 2010 and that 30 patients will be recruited from the North Bristol Lung Centre within 36 months.

10.1 Trial costs

Zoledronic acid costs £195.00 per dose. Drug costs will therefore be a maximum of £5850.00 (15 patients, 2 doses).
0.9% saline placebo will cost a maximum of £500 for 15 patients.
Southmead pharmacy does not currently charge for clinical trial administration.
Calcium and vitamin D supplements will cost £86.00 in total.
Remaining costs to cover include transport expenses to the Cheltenham Imaging Centre (maximum £10,000).
DCE- MRI X 2 for up to 30 patients will be provided by the Cobalt fund as a component of ongoing arrangement to support pleural research.
CT scans have been costed at £ 200, amounting to £12000 for a total of 60 scans.
The total cost for the randomisation service is £2,714.
Biological sample storage and preparation costs are already covered within the infrastructure of the pleural research unit.
Trial stationary costs will be minimal – about £200.00.
Set up and running of the VGEF assay is likely to cost £5000 - £6000 for this small pilot. This requires confirmation.

11.1 Infrastructure

Dr Nick Maskell will oversee conduction of the trial, devoting 1 session per week.
Amelia Dunscombe will devote 2.5 sessions per week to co-ordinating the trial including recruitment, biological sample processing, data collection and ultimately analysis and publication of the trial paper. Her salary is already covered.
A specialist pleural research nurse is fully funded and in post. . Care, both in hospital and the community of patients with indwelling pleural catheters will be a fundamental component of her post. Within the trial, the nurse will be responsible for drug/placebo administration, pleural fluid drainage and volume measurement, venepuncture and collection of pleural fluid samples. In patients with a pleurx catheter in situ, some drainages may be undertaken by a Specialist Lung Cancer nurse from North Bristol NHS Trust trained in the use of indwelling pleural catheters.
Drug/ placebo administration and pleural fluid drainage will take place in the respiratory department procedure room. Alternate

Pleurx catheter drainages will usually be conducted in the patient’s home (usual clinical practice).

12.1 SAE/ SUSAR reporting

The trial team will report all serious adverse events, to the sponsor, in writing within 24 hours using the adverse event reporting form provided by the sponsor.

Definition of SAE:
(a) results in death,
(b) is life-threatening,
(c) requires hospitalisation or prolongation of existing hospitalisation,
(d) results in persistent or significant disability or incapacity, or
(e) consists of a congenital anomaly or birth defect.

The sponsor (North Bristol NHS trust) commits to reporting all suspected unexpected serious adverse reactions (SUSARs) to the MHRA using a CIOMS1 form.
SUSARs that are fatal or life threatening will be reported within 7 days. SUSARs that are not-fatal or life threatening will be reported within 15 days of the sponsor’s knowledge of the event.

The sponsor will keep a written record of all SAEs.

The sponsor will provide an annual safety report to both the MHRA and Ethics committee.

13.1 Ethical issues and risk to patients

Zoledronic acid is associated with infrequent mild side effects and an extremely rare serious side effect. Exclusion criteria and careful biochemical monitoring have been specified to minimise these risks within the trial setting and patients will be fully informed prior to consent being taken.
Patients will be required to visit the hospital regularly for trial purposes. Some of these appointments may occur in the patient’s home. Some patients will travel to Cheltenham on two occasions. Travel expenses will be covered.
Patients in the placebo arm are not expected to experience personal benefit from trial participation.

Up to 12mSv additional radiation exposure is anticipated per patient, the equivalent of a 1 in 1500 chance of a harmful effect. As any harmful effect would not be evident until 5-10 years after

exposure, this is unlikely to be of significance to this population of patients with advanced malignancy.

13.2 Ethical approval

Granted by the South West Research Ethics Committee on 18/05/2009.

13.3 Protocol amendments

1/.03/04/2009
- Inclusion/ exclusion criteria to include age range and clarify issues regarding prescription of hormonal therapy.
- 2.8 Details of calcium, magnesium and phosphate monitoring
- 4.2 Marketing authority of placebo
- 12.1 SAE/ SUSARS.

2/.16/04/2010
-2.2 and 2.7 Reduction of number of drug/placebo doses to 2.
- 2.1-3.1 Inclusion of patients without Pleurx catheters in situ with associated amendment of inclusion criteria, outcome measures and research questions.
- 7.3 Addition of trial CT scans.
- 8.2 Addition of daily dyspnoea VAS
-2.7 Routine dental check up during the run in period added.
-9.1 Extension of trial recruitment and ethical approval to June 2013

2/.11/04/11

- Change of trial co-ordinator details and addition of David Hall and Clare Hooper as key investigators.
- 2.2/2.7 Addition of pleural fluid storage at every drainage for pleurx patients
- 2.3 Addition of Bristol Oncology Centre as a recruiting centre, with the option of opening further centres if recruitment falls below target.
- 2.4 Inclusion/ exclusion criteria to include patients on tarceva for past 3 months and removing need for histological confirmation in patients with proven malignancy elsewhere
- 2.7 clarification regarding use of calcium and vitamin D in patients with hypercalcaemia
- 2.7 addition of specialist lung cancer nurses as people performing drainage of indwelling pleural catheters
- 11.1 addition to specialist lung cancer nurses as performing drainage of indwelling pleural catheters

3/. 15/9/11

- 2.5 Exclusion criteria changed from an estimated creatinine clearance of <50 to an estimated creatinine clearance of <40.

- 2.7Clarification of the method used to calculate the estimated creatinine clearance
- 2.7 and 4.1 Dose reduction for ZA in patients with a creatinine clearance of 40-<50.
- 2.7, 7.1 Change to the CT/MRI schedule to allow the scans for occasional patients to be performed at Southmead Hospital, Bristol under exceptional circumstances.
- 4.1 Include new information on atypical femoral fractures

4/. 19/01/2012

- 2.7 Clarification of what to do in event of unsatisfactory dental check
- 2.7 Removal of visit to hospital on day 7
- 2.7, 6.1, 13.1 Allowing some of the patient visits between the doses of IMP, to be performed at the patients home.

5/. 28/06/2012 (non substantial amendment)

- 6.1 Change to wording regarding centrifuging speeds for biological samples

13.4 Application for ethical approval of protocol amendments
1/. Approved 18/05/2010
2/. Approved 26/05/2011
3/. Approved 21/10/2011
4/. Approved 22/03/2012

Annex A: Daily dyspnoea VAS.

To be presented in 8 booklets of 7 daily VAS tables per patient.

**DAY 1**	**Date:_ _/_ _ _/_ _ _ _**	
(a) On average how breathless have you felt today?<br><br>Not breathless at all — Worst possible breathlessness<br><br>(b) How much has your breathlessness bothered you today?<br><br>No bother at all — Worst possible bother		Any comments?

Annex B

DCE-MRI scan protocol

Scan protocol:

Patients are screened by both the clinical trials team and then experienced radiographers for history of surgery or trauma resulting in implanted metal devices or fragments and excluded from the DCE-MRI scan if these are known or suspected to be present and significant.
Patients are booked on a Philips 1.5 Tesla superconducting magnet system using either an Achieva or Intera magnet. If any patients are particularly claustrophobic they are scanned using a Philips High Field Open System which gives equivalent image quality to the conventional 1.5T systems.

The patient has their venflon inserted into a vein in the antecubital fossa or back of the hand by a suitably qualified radiographer, a radiologist or a member of the clinical trials team. The venflon is left in situ for both examinations. The creatinine level is checked as per CIC policy - ie; if the patient is over 65 years old, has a history of kidney problems or is diabetic.

A full explanation of the MRI scan procedure is given to the patient. They are positioned supine on the scanner couch with respiratory bellows wrapped around the chest. These will improve the quality of the first coronal sequence (STIR) by synchronizing the pulse sequence to the expiration phase of the respiratory cycle.

The Synergy-body coil is then positioned in close proximity to the patients' chest. This is a four element surface coil with both anterior and posterior parts, and is SENSE compatible which is useful to reduce scan time on some sequences.

The patient is instructed to breathe regularly as this will improve image quality, but they will not be asked to hold their breath.

The scan begins with a 3-plane survey scan followed by a reference scan to prepare a valid sensitivity for the later use of SENSE (parallel imaging). An optional STIR coronal sequence follows which is triggered to the respiratory cycle and covers the entire chest from front to back. If this sequence can be tolerated it is used for planning of the main axial acquisition, if not the main axial scan can be planned using the survey images.

The main scan used is the dynamic THRIVE sequence – this is a fat-saturated T1W axial sequence using a SENSE factor of 3 to reduce

scan time. It is run to cover the entire lung fields from just above the apices to just below the bases – usually achieved with around 70 overcontiguous slices of 4mm thickness. Foldover direction is usually in the RL direction but care is taken to ensure there is minimal SENSE artefact overlying the lung fields. It has 20 phases – 1 pre-contrast phase and 19 post-contrast phases if tolerated by the patient. Before running this scan the patient is warned about the contrast injection which will take place between phases 1 and 2. They will not be required to hold their breath.

The contrast consists of 15mls Gadolinium (gadoteric acid) - in pre-filled syringes hand injected at a rate of 1ml per sec.

DCE-MRI PARAMETERS

STIR CORONAL LOCALISER SCAN

FOV 400 x 381mm (may be able to reduce depending on pt size)
RL phase direction
25 slices, 7mm thick with a 1mm gap
TR 1600ms, TE 22ms and TI of 165ms
TSE factor of 8
NSA of 3
Linear profile order
256 x 256 matrix
SENSE – factor 2
Respiratory triggered scan with bellows
3.16 minutes scan time
Real-time reconstruction

THRIVE (3D/T1W/FS) AXIAL SCAN

FOV 400 x 350mm (may be able to reduce the phase matrix depending on pt size)
RL phase direction
4mm slice thickness
Overcontiguous slices – 55 to 70 slices depending on coverage required
SENSE – factor 3
3D/FFE
T1/TFE
SPAIR
TFE factor of 45
Low-high profile order
TR – 8ms/ TE – 4ms
NSA of 2

Flip angle of 10
20 dynamic scans
42 seconds per phase (for 70 slices)
Real-time reconstruction

Annex C

CT scan protocol

Scan Parameters: F.O.V. 300, Collimation 16 x 1.5, Pitch 0.66, KV 120, mAs 200, Slice Thickness 2.00.

Reconstruction parameters: F.O.V. 300, S. Thickness 2.00, Matrix 512, Filter b.

Contrast: Yes Optiray 350 Contrast delay: 30 seconds after the beginning of the injection and scans of the liver for portal venous phase starting at 70s following the onset of injection.
