# Supplementary material for: A Randomised Controlled Trial of Intravenous Zoledronic Acid in Malignant Pleural Disease: A Proof of Principle Pilot Study
Source: PLoS One. 2015 Mar 17;10(3):e0118569. doi: 10.1371/journal.pone.0118569 (PMC4364455; doi:10.1371/journal.pone.0118569)

North Bristol NHS
NHS Trust

NORTH BRISTOL LUNG CENTRE

Consultant: Dr Nick Maskell
Trial Co-ordinator: Dr. Amelia Dunscombe

Respiratory Research unit
Southmead Hospital

Westbury on Trym
Bristol BS10 5NB

Contact numbers:
Trial mobile no (9-5 Mon-Fri): 07810611658
Out of hours emergency contact number: 0117 323 1778

Hospital Switchboard: 0117 950 5050

Patient Information Sheet (Indwelling pleural catheter)

'Bristol Randomised Controlled Trial of Zoledronic Acid in Malignant Pleural disease (Pilot study)'

1. Study Title

This is a research study examining whether a drug that is given as a drip into a vein (Zoledronic acid) can be used to reduce the amount of thickening and fluid produced by the lung lining in patients with cancer and improve symptoms such as breathlessness.
'Randomised controlled trial' means that patients who take part will be randomly allocated to the trial drug or an inactive substance (placebo).
'Pilot study' means that a small group of patients will be involved and a larger study may follow if results from the pilot are promising.

2. Invitation

You have been invited to take part in a research study. Before you decide to take part it is important for you to understand why the research is being done and what it will involve. Therefore please take time to read the following information carefully It is a good idea to discuss taking part in the trial with your family and/or GP before making a decision. If you would like to take time to do this please let us know and a further appointment will be made to see you in about 7

days time. Please feel free to ask us if there is anything that is not clear or if you would like more information.

3.What is the purpose of the trial?
The trial is designed to assess whether a drug that is already used for patients with cancer or bone problems (Zoledronic Acid (ZA)) is helpful in reducing the size of the growth and/or amount of fluid in the lining of your lung and therefore improve your breathing and reduce the number of times fluid needs to be drained in the future. A drug with this effect might make inserting drains unnecessary or reduce the length of time that they need to stay in place for patients in the future.

4.Why have I been chosen?
You have been chosen to consider taking part in this trial because you have cancer and have required the placement of an indwelling pleural catheter to regularly drain fluid from the lining of your lung. We think that patients with the exact problems that you have may benefit from treatment with ZA.

This pilot study is only taking place at Southmead hospital and 30 patients will be asked to participate.

5.Do I have to take part?
It is up to you to decide whether or not to take part. If you do decide to take part you will be asked to sign a consent form which the doctor will also sign and you will then be given a copy to keep for your records along with this information sheet. If you decide to take part, you are free to withdraw at any time without giving a reason. A decision to withdraw, or a decision not to take part will not affect your future medical care outside the trial.

6.What will happen to me if I take part?
(also see figure 1. Page 12)
You will be seen every week for 8 weeks by the trial team. This will usually involve you visiting Southmead hospital, but occasionally a member of the team may be able to visit you at home instead. You will also make 2 trips to the Imaging centre in Cheltenham. All of your travel expenses will be reimbursed or we will book transport for these visits –please let us know which you would prefer.

<u>First 2 weeks</u>

- <u>A blood test will be taken.</u>
- All the fluid will be drained from your lung lining twice a week using the pleural catheter that is already in place. At least one of the weekly drainages will be done at your own home by a specialist nurse, and the other drainage will either be done at home or at Southmead Hospital.
- You will fill in a questionnaire about how you are feeling and how your illness is affecting your life. This takes 10 minutes.
- You will be given a booklet to record how severe your breathlessness is each day.
- You will have a special new scan called a Dynamic MRI scan and an ordinary CT scan. These are done in a single visit at the Cheltenham Imaging centre.
- If you have not seen a dentist in the past 3 months, we will ask you to have a dental check-up (if you do not have a dentist we will arrange this for you at the hospital).
- If you have any dental problems that need treatment or your blood test shows that your kidneys are not working very well, we would not ask you to continue with the study until these have resolved.

<u>Week 3 to 5</u>

- You will be randomly allocated to receive either the active drug (ZA) or a placebo (just salt water). Neither you or any of the doctors or nurses looking after you will know which you were receiving.
- You will be given the drug (ZA) or placebo (salt water) through a drip into a vein in your arm. This takes 15 minutes and will be done in the respiratory department in Southmead. We will take a blood test from the drip before the drug is given.
- You will be asked to take one calcium and vitamin D supplement tablet (chewy) every day after the drug or placebo has been given.
- You will have your fluid drained twice a week. At least one of the weekly drainages will be done at your own home by a specialist nurse, and the other drainage will either be done at home or at Southmead Hospital.
- Once a week you will have a blood test.
- You will continue to note how breathless you feel each day in the booklet provided.
- You will have an ultrasound scan of your chest before your fluid is drained every other week when you attend hospital.

- You will fill in a questionnaire at the end of the 5th week.

Week 6 to 8

- You will be given another dose of the drug or placebo through a drip with a blood test checked before.
- You will have your fluid drained twice a week. At least one of the weekly drainages will be done at your own home by a specialist nurse, and the other drainage will either be done at home or at Southmead Hospital.
- Each week, we will take a blood test.
- You will continue to note how breathless you feel each day in the booklet provided.
- You will have an ultrasound scan of your chest before your fluid is drained every other week when you attend hospital.
- You will fill in a questionnaire at the end of the 8th week.
- You will have one further dynamic MRI scan and CT scan in Cheltenham so that we can see if the drug has changed the appearance of your lung lining.

Samples of your pleural fluid taken from your catheter will be stored to look at the proteins and the drug concentration. This is waste material that has been removed to improve your breathlessness. Blood samples will also be stored.

7/. If I take part, which aspects of my treatment will be 'experimental' or 'extra'?

Zoledronic acid is not usually prescribed for fluid collections around the lung and while the information we have looks promising, we do not know whether it will be beneficial for you and patients like you. Calcium and vitamin supplements, some blood tests, questionnaires, breathlessness scores, some hospital visits to receive the drug and have pleural fluid drained and two MRI scans and two CT scans in Cheltenham (2 visits to Cheltenham in total) will be just for the trial, beyond your usual care. Although this varies between patients, we estimate that you will be required to make up to eight extra visits to hospital for the trial, however these replace visits to your GP practice or appointments when the district nurse would have visited you at home.

The way that we look after your pleural catheter and perform your fluid drainage will be just the same as if you were not taking part in the trial. You received written information about this when your catheter was inserted and we would be happy to give you another copy of this information if it would help you to reach a decision.

Although you will attend hospital for some drainages that would normally have taken place in your home outside the trial, the procedures used will be just the same with no experimental aspects.

8/. About the drug (Zoledronic Acid – ZA)

Zoledronic Acid is a drug that is given through a drip in a small volume of water (100ml). ZA is not a new drug- it is already licensed and very commonly used for patients with bone thinning and cancer that has spread to bones so we know lots about it and how safe it is. Recent research has suggested significant benefits to patients with breast cancer when they receive ZA as well as their standard treatment. When ZA was given to mice with cancer of the lung lining, less fluid was produced. This trial examines whether using ZA in patients like you (who would not routinely receive ZA), might reduce pleural fluid production and ease breathlessness.
The dose used in this trial will depend on your kidney function. It will be given every three weeks for 2 doses. The doses used are already licensed and in common use.

Possible Side effects

Most patients do not experience side effects from the drug.
Table 1. summarises the more common side effects that have been observed.

Table 1.

**Possible side effect**	**How common is it?**	**Notes**
Flu – like symptoms (sometimes with muscle aches and pains).	9 out of every 100 patients.	Usually immediately after the drug is given and can continue for a few days.
Low blood calcium levels requiring treatment.	3 out of every 100 patients.	Easily treated by increasing supplements. If calcium does not improve with supplements, the drug is stopped.
Nausea (sickness)	6 out of every 100 patients treated.	If this happens, anti-sickness tablets are effective in most cases.
Poor appetite	1-2 out of every 100 patients treated.	

Worsening kidney function.	About 2 out of every 100 patients treated.	This problem mainly happens in patients with existing kidney problems who will usually not be included in this trial. The problem is usually reversible by stopping the drug. Kidney function will be monitored carefully for all trial participants.
Anaemia (low blood count).	About 1 out of every 100 patients treated.	Rarely, this requires a blood transfusion. If this happened, the drug would be stopped.
Conjunctivitis (sore eyes)	About 1 out of every 100 patients treated.	
Redness and soreness around the drip site.	About 1 in every 100 patients treated.	This usually settles within a few days of the drug being administered.

Extremely rarely ZA causes a serious problem with the bones of the jaw (osteonecrosis) but we know which patients are most likely to experience this and will not include them in the trial. In order to be absolutely safe, we will ask you to see a dentist for a simple check up in the dental clinic at Southmead hospital (we will arrange this for you). If there is a dental problem that needs treatment we would not ask you to continue with the trial.

Another extremely rare side effect of ZA is an increased risk of fractures of the thigh bone (femur). This is usually seen in patients receiving zoledronic acid on a long term basis for osteoporosis and hence is unlikely to be relevant in this study as you will only receive 2 doses. If you do notice any pain in the groin, hip or thigh during the study, it is important you tell the trial nurses or doctors.

If you think that you are experiencing a side effect from the drug, it is very important that you tell the trial nurses or doctors. Contact numbers are given on pages 1 and 11 of this document.

Pregnancy, breast feeding and conception
As we do not know whether ZA is safe in pregnancy and breast feeding or whether it causes damage to sperm, you should not take part in this study if you are pregnant or breast feeding or are planning a pregnancy or to father a child during or in the six months following the trial.

9. About the placebo (0.9% sodium chloride).

A placebo is an inactive substance given instead of the active drug in trials so that we can be sure that any benefit seen is because of the drug alone. Half of the patients in this trial will receive 100ml of sodium chloride only. This is salt water that is commonly used to dissolve drugs that are given through a drip. It is not associated with any side effects.

10.How will the drug or placebo be administered?

A cannula will be placed in a vein in your arm and ZA or placebo administered as a 'drip' over 15 minutes.
The placing of a cannula can be painful for a few seconds and occasionally it can be difficult and more than one attempt required.
Sometimes cannula insertion can cause a bruise or redness and soreness for a few days after a drug has been administered.
Only highly experienced nurses and doctors will insert your cannula within the trial.

11. About vitamin supplements

You will be provided with calcium and vitamin D supplements combined in one chewy tablet. You will be asked to take one each day for the six weeks of the trial after the drug or placebo is first given.
ZA can reduce your calcium levels if supplements are not given. The supplements are harmless providing your calcium levels are not high.
Your calcium level will be checked each week.

12. About Dynamic MRI scans

This is a special test which involves injecting a dye called gadolinium into a cannula in a vein in your arm (the same way that the trial drug is given) and taking a scan. This takes about 15 minutes. You will be required to lie on a bed which passes under the scanner .The scan is

noisy and although we use an 'open scanner' which is not a tube, some patients still find it a little enclosed. It does not involve any radiation and is very safe.
Two specialist radiology consultants will read your scans, one of whom is a key member of the trial team of doctors.
If a problem that is not related to the lining of the lung is detected on a trial scan, you will be informed and this information will be used to guide your treatment. Any doctors or nurses who are involved in your usual medical care, particularly your GP, will be informed.

13. About CT scans

Your CT scans will be done at the Cheltenham Imaging centre when you visit for the MRI scans and we would aim for you to be at the imaging centre for no more than 2 hours in total (and usually only 1 hour). Having a CT scan involves lying still on a bed which passes through a donut shaped scanner. A dye is injected into your drip. Radiation exposure associated with 2 CT scans carries a risk of harm of about 1 in 1500 (0.0007%) over a ten year period and is not considered significant.

14. What will happen if I am breathless between twice weekly visits?

Any further drainage you need would be performed in your home by our specialist nurses, so you must let us know. We will always measure and record the amount of fluid removed.

15.Will my medical information be kept confidential?

Yes, your medical records will be kept confidential.
In order to arrange and perform the scans at the Cheltenham Imaging Centre, some information about you may be transferred between Southmead and The Cheltenham Imaging Centre.
Your medical records will be examined by key members of the research team (doctors who would usually be involved in your care as well as respiratory doctors and nurses who are co-ordinating the trial) during your involvement in the study. We will also use the results of any chest x-rays and scans that you have needed in the course of your usual care to help with the study. All information will then be recorded and stored on a secure database, accessible only to the research team.

16. What are the possible disadvantages and risks of taking part?

The main risk is of experiencing a side effect to ZA (detailed in section 8). You will be required to visit hospital (including Cheltenham on two occasions) more often that during your normal care and will have more blood tests and cannulas which can be mildly uncomfortable. CT scans are associated with some radiation exposure with the risk of harm from 2 CT scans being about 1 in 1500 (0.0007%) over 10 years.

17. What are the possible benefits of participating in the trial?

If you are allocated the active drug, you may experience an improvement in your breathing with less pleural fluid produced and the possibility of not requiring your catheter any more. We do not know if this will be the case yet.

If you are allocated to the placebo, there will be no direct benefits to your condition.

You will be contributing to our understanding and development of new, better ways to treat your illness which will benefit patients like you in the future.

19. What happens when the research study stops?

After completing 8 weeks of treatment and monitoring within the trial, you will be assessed and will receive the best, evidence based treatment that is currently available. At this time, this does not include ZA.

The study doctor may withdraw you from the trial at any time, if he/she does not feel that it is safe for you to continue. North Bristol NHS trust may also stop the trial before it is due to finish; if this happens, the reasons will be explained to you.

20. What if new information becomes available?

The trial team will continue to review all new research data. If new information that influences the trial becomes available, alterations will be made accordingly. If these will change your treatment or the use of any samples taken from you in any way, you will be contacted with an updated information sheet and asked to sign a further consent form. If you do not wish to continue to participate in light of the new information, you will be withdrawn from the trial and your standard care will not be affected in any way.

21. What if there is a problem?

If you have a concern about any aspect of this study you should ask to speak to the study doctor who will do their best to answer your questions (see number on the front of this sheet). If you remain unhappy and wish to complain formally, you can do this through the NHS complaints procedure. The study doctor will provide you with contact details for the 'Patient advice and liason service' or the complaints manager which can also be obtained from the hospital switchboard.

If you are harmed as a result of your participation in the study due to someone's negligence, North Bristol NHS trust will provide indemnity (cover) and/or compensation via the NHS indemnity scheme.

If you are harmed as a result of your participation in the study, not due to negligence (eg and unexpected side effect of the trial drug), North Bristol NHS trust will sympathetically consider any claim for compensation.

22. Who is organising and funding the research?

North Bristol NHS Trust is sponsoring the research, which means that the trust has overall responsibility for the safe and appropriate conduction of the trial.

Two commercial companies: Novartis pharmaceuticals UK ltd and UK Medical ltd are providing funding for the trial. These companies are not involved in the planning, running or analysis of the trial.

No payment will be made to trial doctors or nurses for including you in the trial.

23. Who has reviewed the trial?

This study has been reviewed and approved by the South West Research Ethics Committee.

24.What will happen to the results of the trial?

When the study has finished and the results have been analysed, they will be published in a medical journal so that other doctors can read them. If you would like us to write to you personally, explaining the study findings, please indicate this on your consent form.

25. What do I need to do?

After reading this sheet, you will be invited to ask questions about the trial. If you would like to take part, we will ask you to sign a consent form. Your GP will be informed that you are taking part.
Please consider checking whether participating in the trial will affect any of your insurance policies and seek advice from the relevant companies if this is a concern.
If you wish to take a few days to consider whether to take part or would like to discuss the trial with your GP, please inform us.
If you decide not to participate, your routine care will not be affected in any way. You can withdraw from the study at any time.
Thank you for taking the time to read this information and consider taking part in the trial.

Chief investigator:
Dr. N. Maskell (Consultant in Respiratory Medicine, North Bristol Lung Centre)

Contact numbers:

Trial mobile number: 07810611658
This phone will be answered 9-5 Monday to Friday by Dr. Amelia Dunscombe or Dr. Nick Maskell. If we are unable to answer the phone immediately, please leave a message and we will phone you back on the same day. You may also leave a message on this number out of working hours and we will phone you back on the following working day.

In an emergency, out of the above working hours, please call 0117 323 5060. This is the number for the respiratory ward. A member of the ward staff will answer the phone, provide emergency advice and put you in touch with Dr. Dunscombe or Dr. Maskell if necessary.

Figure 1: Overview of the Bristol RCT of Zoledronic Acid in malignant pleural disease.

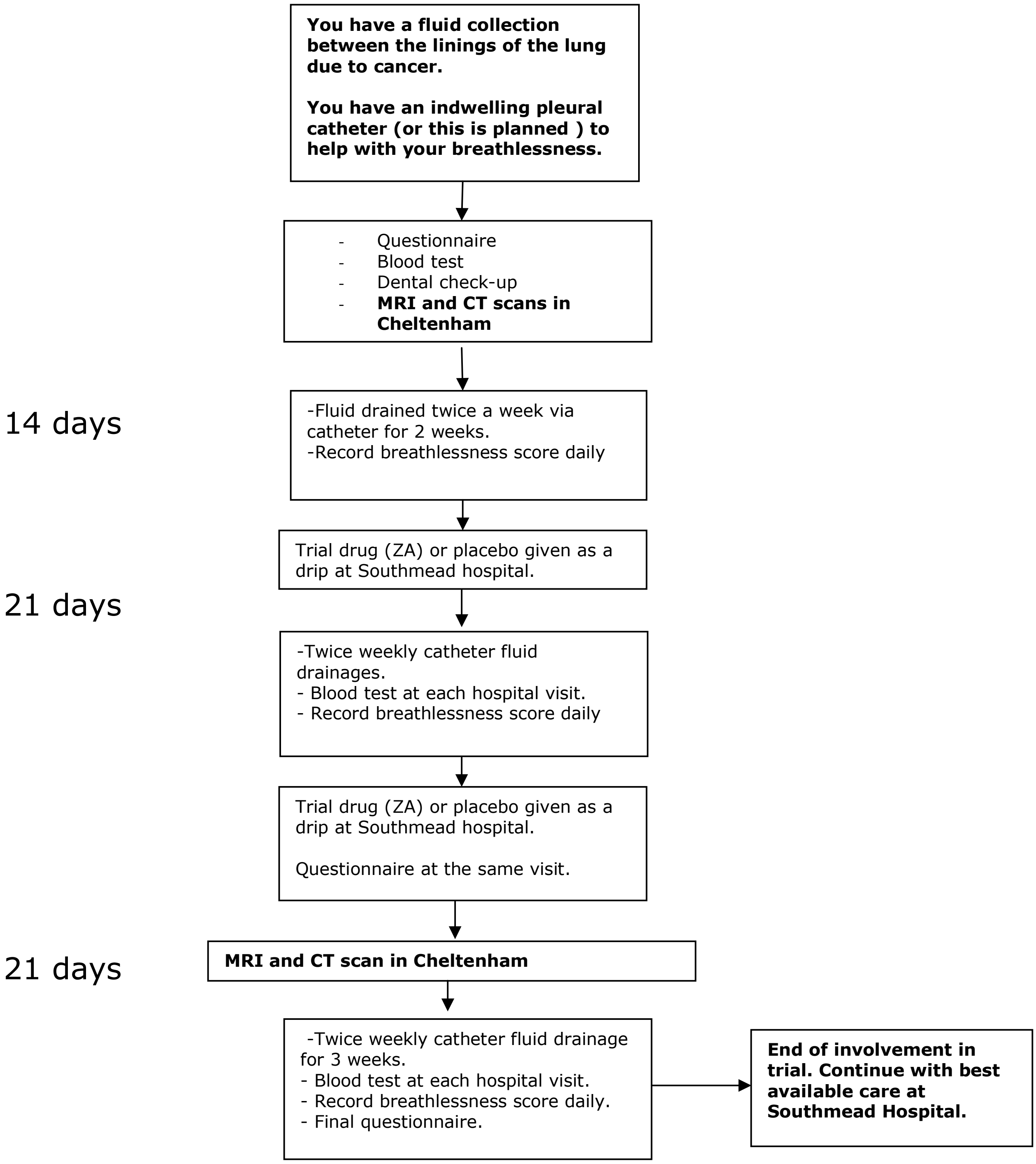

Supplement: S1 File — (PDF) [file pone.0118569.s003.pdf]
