# Supplementary material for: A Randomised Controlled Trial of Intravenous Zoledronic Acid in Malignant Pleural Disease: A Proof of Principle Pilot Study
Source: PLoS One. 2015 Mar 17;10(3):e0118569. doi: 10.1371/journal.pone.0118569 (PMC4364455; doi:10.1371/journal.pone.0118569)
Supplement: S2 File — (PDF) [file pone.0118569.s004.pdf]

Patient consent form

Bristol Randomised Controlled Trial of Zoledronic Acid in Malignant Pleural Disease (Pilot study).

Please initial in each box to indicate that you have read the statement

1/. I confirm that I have read and understand the information sheet (version number…….. dated …./…./……) for the above study and have had the opportunity to ask questions. ☐

2/. I understand that my participation is voluntary and that I am free to withdraw at any time, without giving any reason, without my medical care or legal rights being affected. ☐

3/. I understand that sections of my medical notes will be looked at by members of the research team and may be examined by members of regulatory authorities where it is relevant to my taking part in the research. I give permission for these individuals to have access to my records. ☐

4/. I understand that samples of my blood, and pleural fluid will be stored and used in a future research. The stored samples will not be directly identified to me. ☐

5/. I understand that CT and MRI scans for the trial will be performed at The Cheltenham Imaging Centre. I agree for my information to be shared with individuals at The Cheltenham Imaging Centre for this purpose. ☐

6/. I agree to take part in this study. ☐

7/. I would like my GP (Dr…………………………………..) to be notified about my participation in the study. ☐

____________________	___ ___ ___	______________________
Name of patient	Date	Signature
____________________	___ ___ ___	______________________
Name of researcher	Date	Signature

3 copies, 1 for the patient, 1 for researcher, 1 to be kept with hospital notes.

Would you like to know the results of the trial when they are published? Yes/No (please circle)
