# Supplementary material for: A Randomised Controlled Trial of Intravenous Zoledronic Acid in Malignant Pleural Disease: A Proof of Principle Pilot Study
Source: PLoS One. 2015 Mar 17;10(3):e0118569. doi: 10.1371/journal.pone.0118569 (PMC4364455; doi:10.1371/journal.pone.0118569)
Supplement: S3 File — (DOCX) [file pone.0118569.s005.docx]

**Additional Methodology**

Trial Protocol

The trial protocol can be found as a separate document within the online appendix, which includes full details of the trial conduct and the CT and MRI protocols.

Statistical Analysis Plan

The statistical analysis plan can also be found as a separate document within the online appendix.

MRI analysis Standard Operating Procedure (SOP)

See separate document within the online appendix.

DWI-MRI analysis

ADC quantification from the DWI-MRI scans (with B values of 0 and 1000) was undertaken by an experienced respiratory radiologist, blinded to clinical information and treatment. Three focal regions of restricted diffusion were identified and contoured on the B=1000 dataset at the following levels: the pulmonary venous confluence, the carina and the arch of the aorta. If tumour was not visible at these levels, measurements were taken from the top, middle and bottom of the visible tumour. The 3 measurements were averaged to give a single ADC score for that scan. The absolute change in ADC between the randomisation and week 5 scan were calculated.

CT analysis

The CTs were independently reported by 2 respiratory radiologists using the modified RECIST criteria, with combined consensus evaluation for discrepant values [1 ,2]. Disease >5mm was measured and included in the calculation of the score. For visible disease ≤5mm, a score of 5 was attributed to that level. Two tumour measurements were obtained at each of the following levels: the pulmonary venous confluence, the carina and the arch of the aorta. If tumour was not visible at these levels, measurements were taken from the top, middle and bottom of the visible tumour. These values, along with up to 2 lymph node measurements were summed to give a modified RECIST score.

For those patients who had no CT performed but had an MRI, the MRI was used to calculate the modified RECIST scores (as stipulated in the pre-agreed statistical analysis plan). This was the case for 2 patients in the study.

Biomarker testing

Plasma was sent for a full blood count at each trial visit. The NLR was calculated by dividing the neutrophil count by the lymphocyte count.

Plasma (collected in EDTA) and serum were collected at trial visits. Additionally, for those patients with an IPC or who underwent a pleural aspiration during trial follow up for clinical reasons, pleural fluid (collected in EDTA and serum gel tubes) was collected.

These samples were centrifuged at 1000G for 20mins and the supernatant frozen at -70^o^C for the purpose of subsequent analysis.

*Pleural fluid and blood VEGF quantification*

Commercially available enzyme linked immunoassay (ELISA) kits were used to quantify VEGF levels in the pleural fluid samples (Quantikine © for human VEGF, R&D Systems, Abingdon, UK) and plasma. Samples were processed in duplicate according to manufacturer’s guidelines. The accurate measurement range as determined by the standard solutions is 31.2-2000pg/mL and samples were diluted if necessary.

*MCP-1 quantification*

Commercially available enzyme linked immunoassay (ELISA) kits were used to quantify MCP-1 levels in the plasma (Quantikine © for human CCL2/MCP-1, R&D Systems, Abingdon, UK). Samples were processed in duplicate according to manufacturer’s guidelines. The accurate measurement range as determined by the standard solutions is 31.2-2000pg/mL and samples were diluted if necessary.

IL-6 quantification

Commercially available enzyme linked immunoassay (ELISA) kits were used to quantify IL-6 levels in the plasma (Quantikine © for human IL-6, R&D Systems, Abingdon, UK). Samples were processed in duplicate according to manufacturer’s guidelines. The accurate measurement range as determined by the standard solutions is 3.12-300pg/mL and samples were diluted if necessary.

*Mesothelin quantification*

Commercially available enzyme linked immunoassay (ELISA) kits were used to quantify mesothelin levels in the plasma (Mesomark © for human mesothelin, Fujirebio Diagnostics Inc., Malvern, PA, USA). Samples were processed in duplicate according to manufacturer’s guidelines. The accurate measurement range as determined by the standard solutions is 0-32nM and samples were diluted if necessary.

**Additional Results**

**Table:** Results of Breathlessness and Quality of Life Variables

| Outcome | Number included in analysis | | Baseline value (mean, SD) | | Median (IQR) number of days used to generate summary score | | Summary score  (mean, SD) | | Treatment effect (95% CI) | *p*-value |
| --- | --- | --- | --- | --- | --- | --- | --- | --- | --- | --- |
|  | Placebo arm (n=13) | ZA arm (n=11) | Placebo arm | ZA arm | Placebo arm | ZA arm | placebo arm | ZA arm |  |  |
| ‘On average how much breathlessness have you felt today’ summary VAS score* | 12 | 10 | 22.85 (12.30) | 33.48 (15.16) | 42 (38.5-42) | 42 (42-43) | 21.56 (13.36) | 33.99 (15.03) | 4.16 (-4.70 to 13.03) | 0.333 |
| ‘On average how much has breathlessness bothered you today’ summary VAS score* | 12 | 10 | 23.04 (13.13) | 31.63 (14.59) | 42 (38.5-42) | 42 (42-43) | 20.41 (11.23) | 32.78 (14.39) | 5.45 (-1.44 to 12.35) | 0.113 |
| MRC dyspnoea score* | 12 | 10 | 2.33 (1.07) | 3.10 (1.10) | 42 (41.5-43) | 42 (42-42) | 2.50 (0.97) | 2.92 (1.04) | -0.17 (-0.67 to 0.33) | 0.474 |
| QLQ-C30 overall QOL domain** | 12 | 10 | 67.36 (20.55) | 55.83 (11.82) | 42 (41.5-43) | 42 (42-42) | 60.42 (13.02) | 50.73 (9.49) | -4.05 (-13.04 to 4.94) | 0.352 |
| QLQ-C30 physical functioning domain** | 12 | 10 | 72.78 (16.44) | 62.67 (19.68) | 42 (41.5-43) | 42 (42-42) | 67.64 (13.30) | 61.01 (21.53) | 1.29 (-5.23 to 7.82) | 0.679 |
| QLQ-C30 dyspnoea domain* | 12 | 10 | 27.78 (12.97) | 56.67 (22.50) | 42 (41.5-43) | 42 (42-42) | 29.03 (15.59) | 56.78 (26.29) | 6.46 (-10.86 to 23.77) | 0.439 |
| ESAS*** | 12 | 10 | 15.94 (7.70) | 24.05 (9.91) | 42 (31-43) | 42 (42-42) | 18.61 (8.89) | 25.37 (7.88) | 1.62 (-5.41 to 8.65) | 0.631 |
| *High values for the VAS scores, the EORTC QLQ-C30 dyspnoea score and the MRC dyspnoea score represent worse breathlessness.  **High values for the EORTC QLQ-C30 physical functioning score and the EORTC QLQ-C30 overall quality of life score represent better quality of life.  ***High values for the ESAS score represent worse quality of life  SD= Standard Deviation; IQR= Interquartile Range; 95% CI= 95% confidence interval; MRC= Medical Research Council; VAS= Visual Analogue Scale; EORTC QLQ-C30= European Organisation for Research and Treatment of Cancer Quality of Life Questionnaire; ESAS= Edmonton Symptom Assessment Scale. | | | | | | | | | | |

**Graph of QLQ-C30 Global quality of life (QOL) scores for the 2 treatment arms. The horizontal line with the box represents the median. The box edges represent the lower (25^th^) and upper (75^th^) quartiles. The whiskers represent the lower and upper adjacent values. Outside values are plotted separately.**

**Table: Results of the biomarker studies**

| Outcome | Number included in analysis | | Baseline value (mean, SD) | | Median (IQR) number of days used to generate summary score | | Summary score  (mean, SD) | | Treatment effect (95% CI) | *p*-value |
| --- | --- | --- | --- | --- | --- | --- | --- | --- | --- | --- |
|  | Placebo arm (n=13) | ZA arm (n=11) | Placebo arm | ZA arm | Placebo arm | ZA arm | placebo arm | ZA arm |  |  |
| Blood Mesothelin* | 7 | 6 | 9.0 (11.6) | 7.4 (11.2) | 42 (41-42) | 42 (42-44) | 8.5 (10.7) | 7.5 (10.8) | 0.64 (-1.58 to 2.87) | 0.529 |
| Blood VEGF | 12 | 10 | 128.8 (148.7) | 154.4 (98.6) | 42 (41.5-43) | 42 (42-42) | 156.5 (151.2) | 176.8 (153.1) | -3.69 (-61.65 to 54.27) | 0.894 |
| Blood IL6 | 12 | 9 | 11.0 (5.3) | 18.5 (15.8) | 42 (41-43) | 42 (42-42) | 14.2 (8.5) | 24.0 (31.7) | -0.50 (-17.25 to 16.26) | 0.950 |
| Blood MCP-1 | 12 | 10 | 254.7 (87.0) | 338.5 (293.2) | 42 (41.5-43) | 42 (42-42) | 260.1 (67.4) | 344.1 (329.1) | -3.59 (-65.37 to 58.18) | 0.903 |
| Blood NLR | 12 | 10 | 5.9 (6.3) | 4.0 (3.6) | 42 (41.5-43) | 42 (42-42) | 7.7 (8.7) | 6.6 (10.0) | 1.69 (-2.93 to 6.30) | 0.448 |
| Pleural fluid VEGF | 3 | 2 | 7794 (11392) | 6896 (762) | 42 (41-42) | 41 (40-42) | 7464 (9648) | 9418 (3168) | Insufficient patients to analyse | Insufficient patients |
| Pleural fluid MCP-1 | 3 | 2 | 2615 (839) | 7974 (4548) | 42 (41-42) | 41 (40-42) | 5021 (3813) | 21525 (899) | Insufficient patients to analyse | Insufficient patients |
| SD= Standard Deviation; IQR= Interquartile Range; 95% CI= 95% confidence interval; VEGF= Vascular Endothelial Growth Factor; IL6= Interleukin-6; MCP-1= Monocyte Chemoattractant protein-1; NLR= Neutrophil to Lymphocyte Ratio.  *Mesothelin levels were only tested in patients with mesothelioma | | | | | | | | | | |

**Table:** Results of the pleural fluid volume studies

| Outcome | Number included in analysis | | Baseline value (mean, SD) | | Median (IQR) number of days used to generate summary score | | Summary score/ change from baseline  (mean, SD) | | Treatment effect (95% CI) | *p*-value |
| --- | --- | --- | --- | --- | --- | --- | --- | --- | --- | --- |
|  | Placebo arm (n=13) | ZA arm (n=11) | Placebo arm | ZA arm | Placebo arm | ZA arm | placebo arm | ZA arm |  |  |
| IPC pleural fluid output volume | 4 | 2 | 1076 (685) | 326 (458) | 43 (42- 43) | 42 (41-43) | 732 (103) | 221 (108) | -426 (-986 to 134) | 0.082 |
| USS Depth* | 8 | 6 | 8.5 (12.1) | 1.1 (0.9) | 42 (41-42.5) | 42 (42-42) | 10.4 (13.0) | 1.8 (1.6) | 0.66 (-0.53 to 1.85) | 0.238 |
| CT effusion volume* | 6 | 6 | 623 (1019) | 22 (30) | NA | NA | -34 (49) | -1 (10) | 10.27 (-13.59 to 34.13) | 0.333 |
| SD= Standard Deviation; IQR= Interquartile Range; 95% CI= 95% confidence interval;  * Only patients with no IPC in situ were included in this analysis. | | | | | | | | | | |

**Graph of mean weekly IPC output for the 2 treatment arms. The horizontal line with the box represents the median. The box edges represent the lower (25^th^) and upper (75^th^) quartiles. The whiskers represent the lower and upper adjacent values. Outside values are plotted separately.**

**Table: DCE-MRI outcomes**

| Outcome | Number included in analysis | | Baseline value (mean, SD) | | Change from baseline  (mean, SD) | | Treatment effect (95% CI) | *p*-value |
| --- | --- | --- | --- | --- | --- | --- | --- | --- |
|  | Placebo arm (n=13) | ZA arm (n=11) | Placebo arm | ZA arm | placebo arm | ZA arm |  |  |
| iAUC | 9 | 7 | 113.9 (49.9) | 128.1 (57.6) | 7.3 (46.8) | -20.9 (20.4) | -15.4 (-58.1 to 27.3) | 0.437 |
| Time-to-peak enhancement | 9 | 7 | 433 (73) | 418 (113) | -13 (57) | 31 (134) | 51.3 (-72.3 to 174.9) | 0.372 |
| Initial wash-in slope of the enhancement curve | 9 | 7 | 6.8 (2.6) | 7.3 (3.2) | 1.9 (5.7) | -1.4 (1.7) | -2.9 (-8.5 to 2.8) | 0.278 |
| The maximal contrast enhancement | 9 | 7 | 150.5 (60.9) | 153.3 (70.9) | 19.2 (67.9) | -14.2 (27.2) | -18.3 (-74.1 to 37.5) | 0.477 |
| SD= Standard Deviation; IQR= Interquartile Range; 95% CI= 95% confidence interval; iAUC=The area under the curve measurement in the first 90 seconds | | | | | | | | |

**Table: DWI outcomes**

| Outcome | Number included in analysis | | Baseline value (mean, SD) | | Change from baseline  (mean, SD) | | Treatment effect (95% CI) | *p*-value |
| --- | --- | --- | --- | --- | --- | --- | --- | --- |
|  | Placebo arm (n=13) | ZA arm (n=11) | Placebo arm | ZA arm | placebo arm | ZA arm |  |  |
| ADC Value (mm^2^/sec) | 5 | 3 | 1.74 (0.70) | 1.92 (0.35) | 0.43 (0.30) | 0.22 (0.20) | -0.05 (-3.65-3.55) | 0.896 |
| SD= Standard Deviation; 95% CI= 95% confidence interval; ADC= Apparent Diffusion Coefficient. | | | | | | | | |

**Table: Modified RECIST**

| Outcome | Number included in analysis | | Baseline value (mean, SD) | | Change from baseline  (mean, SD) | | Treatment effect (95% CI) | *p*-value |
| --- | --- | --- | --- | --- | --- | --- | --- | --- |
|  | Placebo arm (n=13) | ZA arm (n=11) | Placebo arm | ZA arm | placebo arm | ZA arm |  |  |
| Modified RECIST score | 11 | 9 | 55.6 (30.3) | 63.9 (34.2) | 3.3 (6.1) | 1.7 (7.4) | -1.79 (-7.90 to 4.33) | 0.539 |
| SD= Standard Deviation; 95% CI= 95% confidence interval. | | | | | | | | |

**SAE details**

| **Study Arm** | **Details of SAE** |
| --- | --- |
| ZA | Hospital attendance for ‘flu like symptoms and myalgia 2 days after IMP administration |
| Placebo | Hospital admission for disease progression and died as an inpatient |
| Placebo | Hospital admission for increasing breathlessness |
| ZA | Hospital admission for increasing breathlessness |
| Placebo | Hospital admission with bowel obstruction |
| ZA | Hospital admission with chest pain and increasing breathlessness |
| Placebo | Hospital admission with general decline and disease progression |
| Placebo | Hospice admission for symptom management |

**Table: protocol deviations**

|  | | **Placebo n (%)**  **(total n=13)** | **Zoledronic Acid n (%)**  **(total n=11)** |
| --- | --- | --- | --- |
| IMP administration | 2 doses given | 11 (85) | 9 (82) |
|  | 1 dose given | 1 (8) | 1 (9) |
|  | 0 doses given | 1 (8) | 1 (9) |
| Dose reduction of IMP required | | 2 (17) | 2 (20) |
| Patient withdrawals post randomisation | Withdrew consent | 0 (0) | 1 (9) |
|  | Clinical decline precluding ongoing study involvement | 2 (15) | 1 (9) |
| Baseline MRI scans | Scan performed and interpretable | 10 (77) | 8 (73) |
|  | Scan performed but uninterpretable | 2 (15) | 1 (9) |
|  | Scan not performed | 1 (8) | 2 (18) |
| Week 5 MRI scans | Scan performed and interpretable | 9 (69) | 7 (64) |
|  | Scan performed but uninterpretable | 2 (15) | 1 (9) |
|  | Scan not performed | 2 (15) | 3 (27) |
| IMP= Investigational Medicinal Product; MRI= Magnetic Resonance Imaging | | | |

**References**

1. Byrne MJ, Nowak AK. Modified RECIST criteria for assessment of response in malignant pleural mesothelioma. Ann Oncol 2004;15(2):257-60

2. Tsao AS, Garland L, Redman M, et al. A practical guide of the Southwest Oncology Group to measure malignant pleural mesothelioma tumors by RECIST and modified RECIST criteria. J Thorac Oncol 2011;6(3):598-601
