# Supplementary material for: A Randomised Controlled Trial of Intravenous Zoledronic Acid in Malignant Pleural Disease: A Proof of Principle Pilot Study
Source: PLoS One. 2015 Mar 17;10(3):e0118569. doi: 10.1371/journal.pone.0118569 (PMC4364455; doi:10.1371/journal.pone.0118569)
Supplement: S4 File — (PDF) [file pone.0118569.s006.pdf]

Standard Operating Procedure for DCE MRI Analysis

- The MRI data will be reported independently by 2 radiologists
- The data obtained will be compared and any discrepancies will be resolved by consensus discussion.
- Each patient underwent 2 trial scans (one pre-treatment and one post-treatment). The scans were performed at 2 centres (Cheltenham and North Bristol). The patients had both of their trial scans done at the same centre.

Regions of Interest

- ‘Regions of interest’ (ROI) will be drawn encompassing all tumour, parietal and visceral pleura at the following levels:
    - Arch of aorta
    - Tracheal carina
    - Pulmonary venous confluence
- If no pleural tumour is visible at these levels, agreed ROIs from the top, middle and bottom of the tumour will be evaluated
- At each ROI, a curve of signal intensity over time will be generated.
- Values at each time point for all three levels will be averaged to generate a global tumour curve.
- Average % enhancement compared with baseline will be calculated for each time point

Analysis of the enhancement curves

- Given different contrast injection techniques at the 2 centres, the following methodology will be used to calculate the ‘baseline’ time:
    - Cheltenham scans
      The ‘baseline’ time will be assumed to be one frame length prior to the first point on the enhancement curve after contrast administration. The tumour signal value at ‘baseline’ will be that obtained from the baseline scan.
    - Bristol scans
      The ‘baseline’ time will be assumed to be the first point on the enhancement curve after the baseline scan has been performed.

- Time from baseline, absolute enhancement and % change in enhancement from baseline will be extracted from the curves at each available time point.

Calculation of AUC

- The trapezium rule will be used to calculate the area under the curve (AUC) and a linear relationship will be assumed between the measured data points.
- The AUC will be interpolated to obtain the value at the necessary time-point after baseline (again, a linear relationship will be assumed between the time-points).

Maximum contrast enhancement (%)

- The durations of the 2 scans for each patient will be calculated from baseline (as defined above) in seconds. The 2 scan durations will be compared and the shorter of the 2 scan durations noted will be called t_{short}.
- In order for the 2 scans to be comparable, the contrast enhancement within t_{short} will be recorded for both scans.
- The maximum contrast enhancement (%) will be calculated using the following equation = (maximal signal intensity-baseline signal intensity)/baseline signal intensity.

Time to peak

- The time taken for the enhancement curve to reach the maximum contrast enhancement ('time-to-peak') will be recorded in seconds from baseline to t_{short}.

Initial slope

- The initial slope will be calculated as the maximal slope (expressed as a change in MR signal intensity in arbitrary units per second) between two consecutive time points (as described by Coolen et al, 2012) from baseline to t_{short}.

PRIMARY OUTCOME:

The change from baseline to week 5 in `initial area under the curve measurement for the first 90 seconds' of the DCE-MRI scan.

SECONDARY OUTCOMES:

DCE MRI
The change from baseline to week 5 post-randomisation in:
1. The initial wash-in slope of the DCE-MRI scan
2. The maximal contrast enhancement of the DCE-MRI scan.
3. The time to peak (seconds) of the DCE-MRI scan.
4. The kep readings from pharmacokinetic analysis of the DCE-MRI scan.
5. The Amp readings from pharmacokinetic analysis of the DCE-MRI scan.
6. The Kel readings from pharmacokinetic analysis of the DCE-MRI scan.

Authorised by:

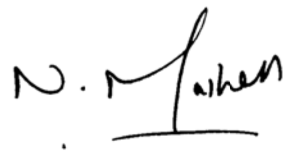

Nick Maskell (Chief Investigator). 2nd July 2014.
