# Supplementary material for: A Randomised Controlled Trial of Intravenous Zoledronic Acid in Malignant Pleural Disease: A Proof of Principle Pilot Study
Source: PLoS One. 2015 Mar 17;10(3):e0118569. doi: 10.1371/journal.pone.0118569 (PMC4364455; doi:10.1371/journal.pone.0118569)
Supplement: S5 File — (PDF) [file pone.0118569.s007.pdf]

Bristol Randomised Controlled Trial of Zoledronic Acid in Malignant Pleural Disease- A Pilot Study.

STATISTICAL CONSIDERATIONS AND ANALYSIS PLAN

The 'Zoledronic Acid Study' is a pragmatic, single centre randomised controlled trial designed to evaluate whether the intravenous bisphosphonate Zoledronic Acid has a role in the management of malignant pleural disease. It was designed as a pilot study to inform a future multi-centre study should the results show promise. The study flow diagram is shown in appendix 1, but in brief, patients were randomly allocated to receive 2 doses of iv Zoledronic Acid or placebo, at randomisation and at 3 weeks post-randomisation, and were followed up for a total of 8 weeks.

RESEARCH QUESTIONS

In patients with malignant pleural disease:

1. Does Zoledronic Acid treatment reduce pleural tumour volume, blood flow and angiogenesis when compared to placebo as measured by serial dynamic contrast enhanced MRI and pleural fluid VEGF-A?
2. Does Zoledronic Acid reduce dyspnoea and improve quality of life compared to placebo?
3. Does Zoledronic Acid reduce pleural tumour progression as measured by CT with volumetric reporting?

Additionally, in patients with a malignant pleural effusion and indwelling pleural catheter:
1. Does Zoledronic Acid reduce pleural fluid accumulation compared to placebo?
2. What is the pleural fluid concentration of Zoledronic Acid at 7, 14 and 21 days following IV administration of a 4mg dose.

OUTCOME MEASURES

Some outcome measures that we will analyse are different from those stated in the original study protocol. This study was devised as a pilot study and hence some of the outcome measures we hoped to evaluate when the study was devised are impractical or impossible to measure given the nature of the recruited population. This statistical analysis plan has been finalised and approved by all the trial investigators before the treatment allocations were un-blinded and before any data analysis was performed.

The trial will report both feasibility and clinical outcomes.

Feasibility outcomes

The feasibility outcomes are:

- The proportion of eligible patients who consented to trial participation
- The proportion of consented patients who were randomised
- The proportion of randomised patients who completed trial follow up
- The reasons for patient exclusions after consent
- The proportion of patients receiving both doses of Investigational Medicinal Product (IMP)
- The proportion of patients requiring a dose reduction of IMP

Clinical outcomes

The clinical outcomes are:

Primary outcomes

1. The change from baseline to week 5 in `initial area under the curve measurement for the first 90 seconds' of the DCE-MRI scan.
2. Mean summary score of the dyspnoea VAS score ('On average how breathless have you felt today?') (Calculations described below) from randomisation to week 6 post-randomisation.

Secondary outcomes

DCE-MRI
The change from baseline to week 5 post-randomisation in:
1. The initial wash-in slope of the DCE-MRI scan
2. The maximal contrast enhancement of the DCE-MRI scan.
3. The time to peak (seconds) of the DCE-MRI scan.
4. The k_{ep} readings from pharmacokinetic analysis of the DCE-MRI scan.
5. The Amp readings from pharmacokinetic analysis of the DCE-MRI scan.
6. The K_{el} readings from pharmacokinetic analysis of the DCE-MRI scan.

DWI-MRI
7. The change from baseline to week 5 post-randomisation in the apparent diffusion coefficient (ADC) of the DWI-MRI scan.

VAS Scores
8. Mean summary score of the dyspnoea VAS score ('How much has your breathlessness bothered you today?') (Calculations described below) from randomisation to week 6 post-randomisation.

Modified RECIST scores
9. The treatment response according to modified RECIST criteria (Complete Response, Partial Response, Stable Disease or Progressive Disease) at week 5 post randomisation
10. The change in the measurable pleural thickening from baseline to week 5 post-randomisation (as assessed using modified RECIST criteria)

CT pleural fluid volumes
11. The change in pleural fluid volume from baseline to week 5 post-randomisation on CT (only in patients with no IPC in situ)

USS
12. The mean summary score (calculations described below) of the overall ultrasound depth from randomisation to week 6 post-randomisation (only in patients with no IPC in situ).

QOL

13. The mean summary score (calculations described below) of the following QLQ-C30 questionnaire domains, from randomisation to week 6 post-randomisation
    a. Global health status
    b. Dyspnoea score
    c. Physical functioning score
14. The mean summary score (calculations described below) of the ESAS-SDS from randomisation to week 6 post-randomisation.

MRC dyspnoea score

15. The mean summary score (calculations described below) of the MRC dyspnoea score from randomisation to week 6 post-randomisation.

Blood biomarkers

16. The mean summary score (calculations described below) of the serum mesothelin levels from randomisation to week 6 post-randomisation.
17. The mean summary score (calculations described below) of the plasma VEGF-A levels from randomisation to week 6 post-randomisation.
18. The mean summary score (calculations described below) of the plasma IL-6 levels from randomisation to week 6 post-randomisation.
19. The mean summary score (calculations described below) of the plasma MCP-1 levels from randomisation to week 6 post-randomisation.
20. The mean summary score (calculations described below) of the serum neutrophil to lymphocyte ratio (NLR) from randomisation to week 6 post-randomisation.

IPC outcomes (IPC patients only)

21. The mean summary score (calculations described below) of the weekly pleural fluid production from randomisation to week 6 post-randomisation.
22. The mean summary score (calculations described below) of the pleural fluid VEGF-A levels from randomisation to week 6 post-randomisation.

Safety outcomes

23. The number of patients experiencing at least one known side effect of Zoledronic Acid (as described in appendix 3 below) from randomisation up to week 6 post-randomisation
24. The number of patients experiencing at least one serious adverse events from randomisation up to week 6 post-randomisation
25. The proportion of patients requiring an increase in calcium, magnesium or phosphate replacement from randomisation up to week 6 post-randomisation

Bioavailability of ZA in pleural space

26. The concentration of Zoledronic Acid in the pleural fluid of patients receiving Zoledronic Acid will be reported descriptively (if funding becomes available for the assay).

GENERAL ANALYSIS PRINCIPLES

Baseline characteristics of all randomised patients will be presented according to treatment group (see appendix 2). For the purposes of analysis, 'randomisation' values will be those recorded prior to but on the day of the first dose of IMP administration, unless otherwise stated.

Analysis will be based on intention-to-treat principles. Patients will be analysed based on the treatment group to which they were randomised, and all randomised patients in whom an outcome is available will be included in the analysis. More information on which participants will be included in each analysis is available in later sections. Data collected to the point at which a patient withdraws from the study will be included in final analysis unless consent for this is withdrawn.

All analyses will adjust for the stratification factors (Malignant cell type; Presence or absence of pleurx catheter; Presence or absence of 'trapped lung'). Continuous outcomes will also adjust for the outcome measured at baseline. Mean imputation will be used to account for baseline variables with missing data.

The number of patients enrolled in the study and the numbers randomised will be presented, and reasons for exclusions will be given. All *p* values will be 2 sided, and the significance level is set at 5%.

Analysis for the feasibility outcomes will be presented as:

1. The number of participants included in the analysis, by treatment group;
2. A summary statistic for the outcome, by treatment group (for example, mean (SD) for continuous outcomes, number(%) for binary outcomes)

Analysis for the clinical outcomes will be presented as:

1. The number of participants included in the analysis, by treatment group;
2. A summary statistic for the outcome, by treatment group (for example, mean (SD) for continuous outcomes, number(%) for binary outcomes)
3. A treatment effect, with a 95% confidence interval and a *p* value

ANALYSIS OF INDIVIDUAL OUTCOMES:

MRI

Patients in the trial undergo 2 trial MRI scans (one at baseline and one at 5 weeks post-randomisation). Only patients who had the week 5 scan will be included in the analysis. Two radiologists will independently report the MRI scans. If there is >20% difference between the 2 reports, a consensus will be reached by discussion. If <20% difference, the results from radiologist 1 will be used for the analysis.

The change in the MRI measurements from baseline to week 5 will be compared between the 2 treatment groups using a linear regression model, adjusting for stratification factors and baseline measurable pleural thickening.

VAS Scores

Patients in the trial have completed two daily VAS scores from 1 week prior to randomisation until 6 weeks post randomisation:

a) ‘On average how breathless have you felt today?’
b) ‘How much has your breathlessness bothered you today?’

The VAS scores will be measured according to the standard operating procedure by 2 independent assessors.

A graph of the daily VAS scores over time from randomisation until week 6 post-randomisation will be produced, and the area under the curve (AUC) will be calculated using the trapezium rule.

The calculated AUC will be divided by the number of days after randomisation that the patient remained in the study to account for patients with different durations of follow-up. Patients will be considered in the study until the final day in which a VAS score was completed. This will give a mean summary score for each patient.

The mean summary scores for patients in the ZA and placebo arms will be compared by means of a linear regression model, adjusting for stratification factors and the patient’s mean baseline VAS score (calculated by averaging the VAS score measurements from the 7 days ‘run-in’ period prior to randomisation).

All patients who have provided at least one VAS score after randomisation will be included in the analysis. Reasons for exclusions will be given. The median number of time points used for calculation of the AUC per patient will be reported.

Modified Recist Criteria

Patients undergo 2 sets of scans during the trial (one at baseline and one at 5 weeks later). For patients who underwent both CT scans, these will be reported using the modified RECIST criteria. In those who did not receive both CT scans, but did have both MRI scans, the MRIs will be used instead to calculate the modified RECIST scores. Patients who did not have any 5 week scans will not be included in the analysis. Reasons will be given for non-inclusions.

Two radiologists will independently report the scans using the modified RECIST criteria (Tsao 2011), to give an overall pleural thickness measurement for each scan. The change in measurable pleural thickening from baseline to week 5 will be compared between the 2 treatment groups, using a linear regression model, adjusting for stratification factors and baseline measurable pleural thickening.

A treatment response (complete response, partial response, stable disease or progressive disease) from baseline to week 5 will be assigned to each patient according to published definitions (Byrne et al, 2004). If the data allows, ordinal logistic regression will be used to compare treatment response between the 2 treatment groups, adjusting for stratification factors. Alternatively, the data will be presented descriptively.

CT pleural fluid volumes

In those patients with no IPC in situ, volumetric analysis will be undertaken to quantify the volume of pleural fluid within the pleural cavity on the CT scans. If there is >20% difference between the 2 reports, a consensus will be reached by discussion. If <20% difference, the mean of the 2 reports will be used for the analysis.

The absolute change in pleural fluid volume from baseline to week 5 will be compared between the 2 treatment groups, using a linear regression model, adjusting for stratification factors and baseline pleural fluid volume.

USS

Each time the patient undergoes a thoracic ultrasound, the maximum effusion depth will be measured at 3 sites (the mid-clavicular, mid-axillary and mid-scapular lines) and summed to produce an overall depth measurement.

The overall ultrasound effusion depths measured at randomisation, 3 weeks post-randomisation, and 6 weeks post-randomisation will be used to produce a graph of effusion depth over time. The area under the curve (AUC) will be calculated using the trapezium rule.

The calculated AUC will be divided by the number of days post randomisation the patient remained in the study to account for patients with different durations of follow-up. Patients will be considered in the study until the final day in which an ultrasound effusion depth is available. This will give a mean summary score for each patient.

The mean summary scores for patients in the ZA and placebo arms will be compared by means of a linear regression model, adjusting for stratification factors and the ultrasound effusion depth at randomisation.

All patients who have at least one thoracic ultrasound performed after their randomisation scan and do not have an indwelling pleural catheter in situ will be included in the analysis. Reasons for exclusions will be given. The median number of time points used for calculation of the AUC per patient will be reported.

QOL

(a) QLQ-C30

A score out of 100 will be calculated for each of the following sub-scales each time a patient completes a QOL questionnaire:

- Global health status/QOL
- Physical functioning
- Dyspnoea.

Each sub-scale will be analysed separately.

The sub-scale scores recorded at randomisation, 3 weeks post-randomisation, and 6 weeks post-randomisation will be used to produce a graph of the sub-scale score over time. The area under the curve (AUC) will be calculated using the trapezium rule.

The calculated AUC will be divided by the number of days post randomisation the patient remained in the study to account for patients with different durations of follow-up. Patients will be considered in the study until the final day in which a sub-scale score was recorded. This will give a mean summary score for each patient.

The mean summary scores for patients in the ZA and placebo arms will be compared by means of a linear regression model, adjusting for stratification factors and the sub-scale score at randomisation.

All patients who have least one sub-scale score recorded after randomisation will be included in the analysis. Incompletely completed questionnaires where calculation of the sub-scale score is not possible will be excluded. Reasons for exclusions will be given. The median number of time points used for calculation of the AUC per patient will be reported.

(b) Edmonton Symptom Assessment Scale (ESAS)

The ESAS questionnaire asks patients to score 10 symptoms from 0 to 10 (10 being the worst). The sum of these scores gives a score out of 100 known as the 'Symptom Distress Score' (ESAS-SDS)

The ESAS-SDS scores recorded at randomisation, 3 weeks post-randomisation and 6 weeks post-randomisation will be used to produce a graph of ESAS-SDS score over time. The area under the curve (AUC) will be calculated using the trapezium rule.

The calculated AUC will be divided by the number of days after randomisation that the patient remained in the study to account for patients with different durations of follow-up. Patients will be considered in the study until the final day in which an ESAS-SDS score was recorded. This will give a mean summary score for each patient.

The mean summary scores for patients in the ZA and placebo arms will be compared by means of a linear regression model, adjusting for stratification factors and the ESAS-SDS score at randomisation.

All patients who have least one ESAS-SDS score recorded after randomisation will be included in the analysis. Incompletely completed questionnaires will be excluded. Reasons for exclusions will be given. The median number of time points used for calculation of the AUC per patient will be reported.

Change in MRC dyspnoea score

The MRC dyspnoea scores recorded at randomisation, 3 weeks post-randomisation, and 6 weeks post-randomisation will be used to produce a graph of the MRC dyspnoea score over time. The area under the curve (AUC) will be calculated using the trapezium rule.

The calculated AUC will be divided by the number of days after randomisation that the patient remained in the study to account for patients with different durations of follow-up. Patients will be considered in the study until the final day in which an MRC dyspnoea score was recorded. This will give a mean summary score for each patient.

The mean summary scores for patients in the ZA and placebo arms will be compared by means of a linear regression model, adjusting for stratification factors and the MRC dyspnoea score at randomisation.

All patients who have least one MRC dyspnoea score recorded after randomisation will be included in the analysis. Reasons for exclusions will be given. The median number of time points used for calculation of the AUC per patient will be reported.

Blood Biomarkers

Levels of the following biomarkers will be measured at randomisation, 3 weeks post-randomisation and 6 weeks post-randomisation:

a. Serum mesothelin levels
b. Plasma VEGF levels
c. Plasma IL-6 levels
d. Plasma MCP-1 levels
e. Neutrophil Lymphocyte Ratio (NLR)

Each biomarker will be analysed separately.

A graph of the biomarker concentration over time will be produced, and the area under the curve (AUC) will be calculated using the trapezium rule.

The calculated AUC will be divided by the number of days after randomisation that the patient remained in the study to account for patients with different durations of follow-up. Patients will be considered in the study until the final day in which a biomarker concentration is available. This will give a mean summary score for each patient.

The mean summary scores for patients in the ZA and placebo arms will be compared by means of a linear regression model, adjusting for stratification factors and the biomarker concentration at randomisation.

All patients who have least one biomarker concentration after randomisation will be included in the analysis. Reasons for exclusions will be given. The median number of time points used for calculation of the AUC per patient will be reported.

Pleural fluid VEGF-A levels (IPC patients only)

For patients with an IPC in situ, pleural fluid VEGF-A levels will be measured at randomisation and then every week after randomisation until week 6.

A graph of pleural fluid VEGF-A concentration over time will be produced and the area under the curve (AUC) will be calculated using the trapezium rule.

The calculated AUC will be divided by the number of days after randomisation that the patient remained in the study to account for patients with different durations of follow-up. Patients will be considered in the study until the final day in which a pleural fluid VEGF-A concentration is available. This will give a mean summary score for each patient.

The mean summary scores for patients in the ZA and placebo arms will be compared by means of a linear regression model, adjusting for stratification factors and the VEGF-A concentration at randomisation.

All patients who have least one VEGF-A concentration measured after randomisation will be included in the analysis. Reasons for exclusions will be given. The median number of time points used for calculation of the AUC per patient will be reported.

IPC pleural fluid output volume (IPC patients only)

For each week after randomisation, a total pleural fluid output volume will be calculated by adding the drainage volumes from all the drainages performed during that week together.

A graph of weekly drainage volume over time from randomisation to week 6 will be produced, and the area under the curve (AUC) will be calculated using the trapezium rule.

The calculated AUC will be divided by the number of days after randomisation that the patient remained in the study to account for patients with different durations of follow-up. Patients will be considered in the study until the final day in which a weekly pleural fluid output volume was calculated. This will give a mean summary score for each patient.

The mean summary scores for patients in the ZA and placebo arms will be compared by means of a linear regression model, adjusting for stratification factors and the weekly pleural fluid output volume calculated for the 'run-in' week immediately prior to randomisation.

All patients who have pleural fluid output volume data for at least one week after randomisation will be included in the analysis. Reasons for exclusions will be given. The median number of time points used for calculation of the AUC per patient will be reported.

SUB GROUP ANALYSIS

If there are sufficient numbers to allow meaningful subgroup analyses, the following subgroups will be examined (analyses will be descriptive only):

- Chemotherapy or endocrine therapy initiated during trial vs. no systemic therapy initiated.
- Mesothelioma vs no mesothelioma
- Presence or absence of 'trapped lung' (defined as incomplete lung re-expansion on plain chest x-ray following initial drainage to dryness at thoracoscopy, ward based intercostal drain or indwelling catheter placement).
- Pleurx catheter in situ or no IPC in situ

The subgroup analyses will be performed for the primary outcome measures and CT criteria.

Appendix 1: Study overview

30 patients with symptomatic malignant pleural effusions and or malignant pleural thickening.

Baseline tests and run-in period
1/. - Baseline pleural fluid (if available) and plasma VEGF level. Baseline renal function with creatinine clearance and calcium profile. DCE-MRI and CT thorax (Cheltenham Imaging Centre). MRC dyspnoea score. Q of L questionnaires (EORTC and ESAS).

14 day run- in period All patients dyspnoea VAS daily. Pleurx catheter patients, twice weekly drainage to dryness with record of fluid volume drained in 14 days prior to randomisation with pleural fluid stored for VEGF analysis at each drainage.

Randomisation with stratification for:
1/. Malignant cell type.
2/. Presence or absence of pleurx catheter
3/. Presence or absence of 'trapped lung'.

2/. Intervention group:
4mg of IV Zoledronic acid in 100ml Nsaline over 15 mins.

Patients, clinical and research team blinded to allocation.

2/. Placebo group:
100ml Nsaline IV over 15 mins.

3/. **All patients**
Daily for 21 days – dyspnoea VAS. Every 7 days for 21 days: Blood for renal function and plasma stored for VEGF. Pleural aspiration and fluid stored for VEGF and ZA level at 14 days when possible (+ when therapeutic aspiration required).
Q of L and MRC dyspnoea score at 21 days.
Pleurx catheter patients Drainage to dryness twice weekly for 21 days with careful drainage volume recording and twice weekly fluid storage for VEGF and ZA levels.

21 days

3/. **All patients**
Daily for 21 days – dyspnoea VAS. Every 7 days for 21 days: Blood for renal function and plasma stored for VEGF. Pleural aspiration and fluid stored for VEGF and ZA level at 14 days when possible (+ when therapeutic aspiration required).
Q of L and MRC dyspnoea score at 21 days.
Pleurx catheter patients Drainage to dryness twice weekly for 21 days with careful drainage volume recording and twice weekly fluid storage for VEGF and ZA levels.

Steps 2 and 3 repeated over a further 21 day period.
Renal function and serum calcium checked prior to each dose of drug/placebo with appropriate dose reduction as per manufacturer's guidelines.
DCE – MRI and CT thorax (Cheltenham Imaging centre) 10- 14 days following second dose of drug/placebo.

Final assessment at 42 days post randomisation

Steps 2 and 3 repeated over a further 21 day period.
Renal function and serum calcium checked prior to each dose of drug/placebo with appropriate dose reduction as per manufacturer's guidelines.
DCE – MRI and CT thorax (Cheltenham Imaging centre) 10- 14 days following second dose of drug/placebo.

Primary outcome (compared between placebo and intervention groups)
1/. Change in gadolinium uptake and washout rate on DCE-MRI
2/. Change in dyspnoea VAS score.

Secondary outcomes (compared between placebo and intervention groups):
1/. Disease progression as measured by volume of pleural thickening on CT scan
2/. Disease progression as measured by volume of pleural thickening on DCE-MRI scan.
3/. For patients with a pleurx catheter in situ, change in fluid volume production between week 2 of run in period and each week post intervention or placebo.
4/. For patients with pleurx catheter in situ, spontaneous pleurodesis rate.

5/. Change in pleural fluid and plasma VEGF between baseline and each aspiration following intervention or placebo (with correlation to fluid drainage in pleurx patients).

6/. Change in volume of pleural fluid on CT.

Appendix 2: Baseline patient characteristics

The following baseline patient characteristics will be tabulated and compared between the 2 treatment arms:

- Proportion of male patients
- Age (years)
- Underlying tumour type
- Mode of diagnosis of pleural malignancy (cytology/histology/confirmation from elsewhere)
- Length of time from diagnosis with MPE to trial entry (days)
- % having received previous chemotherapy for this malignancy
- Side of effusion
- Randomisation MRC dyspnoea score
- Drug history (Specifically use of steroids)
- % having had a previous pleurodesis
- % having had previous thoracic surgery
- % with IPC in situ
- % with trapped lung
- % requiring dental treatment prior to study involvement
- Baseline ’week mean VAS score’ calculated for the week prior to randomisation
- Randomisation ESAS-SDS score
- Randomisation QLQ-C30 scores for global quality of life, dyspnoea and physical functioning
- Randomisation summed effusion depth on thoracic USS

Baseline blood tests will be presented, comparing the 2 treatment arms:

- Total white cell count
- Haemoglobin
- Platelet count
- estimated creatinine clearance
- Corrected calcium
- Phosphate
- Magnesium
- Neutrophil to lymphocyte ratio

Deviations to the trial protocol will be described and reasons for deviations described, with particular focus on:

- IMP administration (dose or timings)
- Patient withdrawals
- Trial CT and MRI scans

The numbers of patients who experienced the known side effects of Zoledronic Acid will be tabulated for the 2 treatment arms (see appendix 3).

Appendix 3: Main side effects of Zoledronic Acid to be reported.

**Side Effect**	**Definition**
Haematological disorder	Fall in Hb, WCC &/or platelets requiring intervention
Allergic reaction	Hypersensitivity reaction, rash or injection site reaction
Headache	New or worsening headache
Fever or 'Flu like syndrome	As defined in the SMPC
Myalgia or Arthralgia	New or worsening symptoms
Electrolyte disturbance	Change in electrolytes requiring intervention
Renal disturbance	Increase in serum creatinine by >44 µmol/l
Eye disorder	New or worsening conjunctivitis, blurred vision or scleritis
Gastrointestinal symptoms	New or worsening nausea or vomiting, abdominal pain or change in bowel habit
Osteonecrosis of the jaw	Clinical definition
Other	Any other reported side effect deemed important

Analysis plan agreed by:

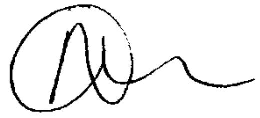

Amelia Clive

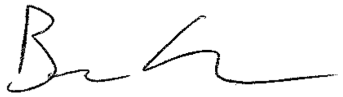

Brennan Kahan

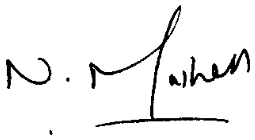

Nick Maskell

25th April 2014.
